# Parameterized syncmer schemes improve long-read mapping

**DOI:** 10.1371/journal.pcbi.1010638

**Published:** 2022-10-28

**Authors:** Abhinav Dutta, David Pellow, Ron Shamir

**Affiliations:** 1 Computer Science and Engineering, Indian Institute of Technology Patna, Patna, India; 2 Blavatnik School of Computer Science, Tel-Aviv University, Tel-Aviv Israel; Harvard, UNITED STATES

## Abstract

**Motivation:**

Sequencing long reads presents novel challenges to mapping. One such challenge is low sequence similarity between the reads and the reference, due to high sequencing error and mutation rates. This occurs, e.g., in a cancer tumor, or due to differences between strains of viruses or bacteria. A key idea in mapping algorithms is to sketch sequences with their minimizers. Recently, syncmers were introduced as an alternative sketching method that is more robust to mutations and sequencing errors.

**Results:**

We introduce parameterized syncmer schemes (PSS), a generalization of syncmers, and provide a theoretical analysis for multi-parameter schemes. By combining PSS with downsampling or minimizers we can achieve any desired compression and window guarantee. We implemented the use of PSS in the popular minimap2 and Winnowmap2 mappers. In tests on simulated and real long-read data from a variety of genomes, the PSS-based algorithms, with scheme parameters selected on the basis of our theoretical analysis, reduced unmapped reads by 20-60% at high compression while usually using less memory. The advantage was more pronounced at low sequence identity. At sequence identity of 75% and medium compression, PSS-minimap had only 37% as many unmapped reads, and 8% fewer of the reads that did map were incorrectly mapped. Even at lower compression and error rates, PSS-based mapping mapped more reads than the original minimizer-based mappers as well as mappers using the original syncmer schemes. We conclude that using PSS can improve mapping of long reads in a wide range of settings.

## Author summary

Popular long-read mappers use minimizers, the minimal hashed *k*-mers from overlapping windows, as alignment seeds. Recent work showed that syncmers, which select a fixed set of *k*-mers as seeds, are more likely to be conserved under errors or mutations than minimizers, making them potentially useful for mapping error-prone long reads. We introduce a framework for creating syncmers, that we call parameterized syncmer schemes, which generalize those introduced so far, and provide a theoretical analysis of their properties. We implemented parameterized syncmer schemes in the minimap2 and Winnowmap2 long-read mappers. Using parameters selected on the basis of our theoretical analysis we demonstrate improved mapping performance, with fewer unmapped and incorrectly mapped reads on a variety of simulated and real datasets. The improvements are consistent across a broad range of compression rates and sequence identities, with the most significant improvements for lower sequence identity (high error or mutation rates) and high compression.

This is a *PLOS Computational Biology* Methods paper.

## Introduction

As the volume of third-generation, long-read sequencing data increases, new computational methods are needed to efficiently analyze massive datasets of long reads. One of the most basic steps in analysis of sequencing data is mapping reads to a known reference sequence or to a database of many sequences. Several long-read mappers have been proposed [1, 2], with minimap2 [3] being the most popular. minimap2 is a multi-purpose sequence mapper that uses sequence minimizers as alignment seeds. Minimizers, the minimum valued *k*-mers in windows of *w* overlapping *k*-mers of a sequence, are used to sketch sequences. They have greatly improved the computational efficiency of many different sequence analysis algorithms (e.g. [4], [5], [6]). A key criterion in evaluating minimizer schemes is their *compression rate*, the number of *k*-mers in the sequence divided by the number of *k*-mers selected. Achieving higher compression rate is desirable, as fewer seeds are used.

Recent work has shown that minimizers are less effective under high error or mutation rates [7]. Motivated by this observation, Edgar [7] recently introduced a novel family of *k*-mer selection schemes called *syncmers*. Syncmers are a set of *k*-mers defined by the position of their minimum *s*-long substring (*s*-minimizer). Syncmers constitute a predetermined subset of all possible *k*-mers and, unlike minimizers, they are defined by the sequence of the *k*-mer only and do not depend on the rest of the window in which they appear. Syncmers are therefore more likely to be conserved under mutations than minimizers. This difference is crucial in long reads, which have much higher error rate than short reads [8]. Another key difference between syncmer and minimizer schemes is that the latter guarantee, for any input parameter *w*, selection of a *k*-mer in every window of *w* consecutive *k*-mers (this is called a *window guarantee*), while syncmers do not. For longer reads with a higher error rate, *conservation* of the selected *k*-mers becomes more important than the window guarantee, especially when there are also mutations. For example, it was shown that with 90% identity between aligned sequences, only about 30% of the positions on the sequence will overlap a conserved minimizer in minimap2 [7].

Edgar defined several syncmer variants, including the families of *open syncmers*, whose *s*-minimizer appears at one specific position, and *closed syncmers*, whose *s*-minimizer appears at either the first or the last position in the *k*-mer [7]. He computed the properties of a range of syncmer schemes and used them to choose a scheme with a desired lower bound on compression rate. Shaw and Yu [9] recently formalized the notions of the conservation of selected positions and their clustering along a sequence, and provided a broader theoretical analysis.

In this work we generalize Edgar’s syncmer schemes to multiple arbitrary *s*-minimizer positions. We call these *parameterized syncmer schemes* (PSS; we use this acronym for both singular and plural). The parameters are the possible indices of the *s*-minimizer in a selected *k*-mer, and an *n*-parameter scheme uses *n* such indices. An example is a 3-parameter scheme that selects any 15-mer with the minimum 5-mer appearing at position 1, 5, or 9. PSS have the advantage of allowing for a larger range of compression rates than syncmers by varying the number of parameters used, *k*-mer length, and *s*-minimizer length.

Two important related features of a scheme are robustness to sequence changes and the distances between selected positions. The conservation of a scheme is the fraction of positions in a sequence covered by selected *k*-mers that are unchanged after the sequence is mutated. The spread of a scheme is a vector of probabilities, where *P*(*α*) is the probability of selecting at least one position in a window of length *α*. Recently, Shaw and Yu [9] obtained expressions for the conservation of open and closed syncmers as a function of spread and implemented these syncmers in minimap2. Here we extend the theoretical analysis by presenting a general recursive expression for the spread of any PSS, including downsampling. These expressions allow for the calculation of the conservation of any PSS. We analyse properties of PSS, including their conservation and spread, and determine which schemes perform well in terms of these properties for a given compression rate through theoretical analysis and empirical testing. Additionally, while closed syncmers are a subset of 2-parameter PSS, our analysis demonstrates that they are not the optimal 2-parameter scheme under realistic mutation rates, and it enables us to instead select the best 2-parameter scheme in terms of conservation and spread.

We introduced PSS into two leading long-read mappers: the latest release of minimap2 [10] and Winnowmap2 [11], where PSS parameters were selected based on our theoretical analysis, and measured the performance compared to the original algorithms on both simulated and real long-read data. The PSS increased the number of mapped reads across a large range of compression rates, resulting in 20–60% fewer unmapped reads at high compression. Even at lower compression, the PSS mappers had 2–15% fewer unmapped reads. The PSS versions used less memory but had longer mapping times than the original mappers for the same compression. The most marked improvements were observed when identity of the mapped reads and reference sequences were low. With identity of 65% and 75% and medium compression, PSS mappers had 50–60% fewer unmapped reads and still had 8–13% fewer incorrectly mapped reads. When using the 2-parameter PSS with best conservation and spread according to our theoretical analysis in minimap2 in comparison to minimap2 using Edgar’s closed syncmers, the former showed a consistent improvement of up to 7% fewer unmapped reads.

Our contribution in this work is thus three-fold: (1) We introduce PSS, generalizing existing syncmer schemes. (2) We provide a theoretical analysis of PSS properties. The analysis enables us to choose the optimal scheme in terms of conservation and spread for particular mutation and compression rates. (3) We provide implementations of minimap2 and Winnowmap2 that use PSS and demonstrate their improved mapping performance compared to the original minimizer versions and to using closed syncmers. Unlike previous work, our mapping implementations also enable downsampling, so that any desired compression rate can be achieved, and on the other hand they have the option to provide a window guarantee.

The paper is structured as follows: we first provide background, definitions, and terminology; the next section provides theoretical analysis of PSS and describes the practical implementation of PSS and their integration into minimap2 and Winnowmap2; the following section presents experimental results of the original and PSS-modified mappers; the final section discusses the results and future work.

## Definitions and background

### Basic definitions and notations

For a string *S* over the alphabet Σ, a *k-mer* is a *k*-long contiguous substring of *S*. The *k*-mer starting at position *i* is denoted *S*[*i*, *i* + *k* − 1] (string indices start from 1 throughout). We work with the nucleotide alphabet: Σ = {*A*, *C*, *G*, *T*}.

***k*-mer order**: Given a one-to-one hash function on *k*-mers $o:\Sigma^{k}\to R$, we say that *k*-mer *x*_1_ is less than *x*_2_ if *o*(*x*_1_) < *o*(*x*_2_). Examples include lexicographic encoding or random hash. We will write instead *x*_1_ < *x*_2_ when *o* is clear from the context. In this work we use a random order unless otherwise noted.

### Selection schemes

#### Selection scheme

A *selection scheme* is a function from a string to the indices of positions in it $f:\Sigma^{*}\to P\left(N\right)$ (P represents the power set). The scheme implicitly selects the *k*-mers starting at these positions. For a string *S* ∈ Σ*, *f*_*k*_(*S*) = {*i*_1_, *i*_2_, …, *i*_*n*_} is the set of start indices of the *k*-mers selected by the scheme.

#### Minimizers

**Fig 1:**
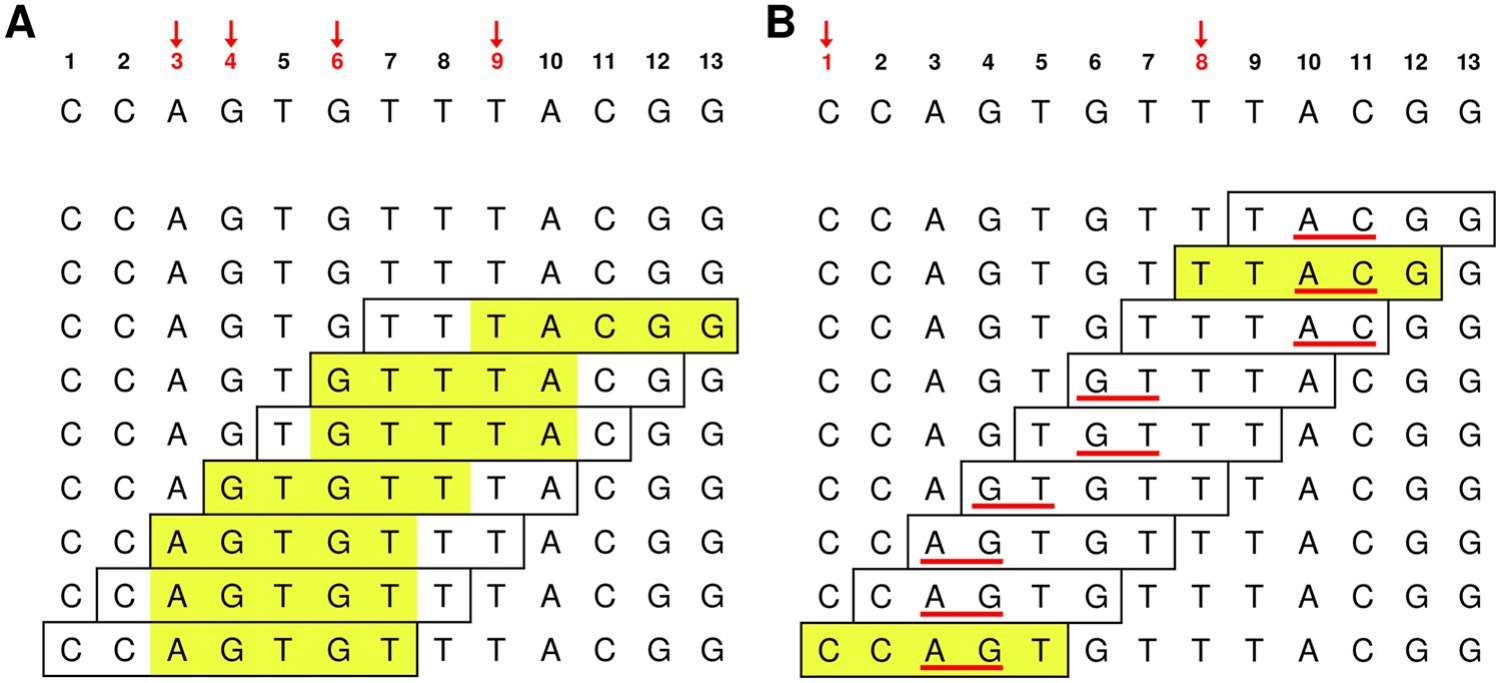
Minimizer and syncmer schemes. In both examples the lexicographic order is used, and only forward *k*-mers are considered. The underlying sequence is shown at the top. By convention the leftmost position is selected in the case of a tie. **(A)** Minimizers. Here *w* = 3 and *k* = 5, so the minimizer is the least 5-mer in every window of length 7. The minimizer of each window is highlighted in yellow; **(B)** Syncmers. Here we show the 1-parameter syncmer with *k* = 5, *s* = 2 and *x*_1_ = 3, $S_{5,2,lex}\left(3\right)$. It selects 5-mers if their 2-minimizer appears at position 3. The 2-minimizer in each 5-mer is underlined in red, and selected *k*-mers are highlighted in yellow. The start positions of the *k*-mers in the underlying sequence that are selected by each scheme appear in red and are marked with red arrows at the top. Sequence positions 6–7 constitute a gap in the syncmer selection as they are not covered by any selected *k*-mer.

A *minimizer scheme* is a selection scheme that chooses the position of the minimum value *k*-mer in every window of *w* consecutive *k*-mers in *S*:

$$\begin{array}{c} M_{k,w,o}\left(S\right)=\overset{|S|-w-k+2}{\underset{j=1}{⋃}}\{\underset{i:i\in [j,j+w-1]}{\text{argmin}}\;S\left[i,i+k-1\right]\} \end{array}$$ (1)

where the minimum is according to *k*-mer ordering *o*. By convention, ties are broken by choosing the leftmost position. An example of a minimizer selection scheme is shown in Fig 1A. By construction, minimizers select a *k*-mer in every window of *w* *k*-mers. This property is called a *window guarantee*.

#### Syncmers

A *syncmer* [7] is a selection scheme that selects a *k*-mer if its minimum *s*-mer is in a particular position or positions. A *closed syncmer* selects *k*-mers whose smallest *s*-mer is at the start or end of the *k*-mer, and an *open syncmer* select *k*-mers whose smallest *s*-mer is at the start only. Note that, unlike minimizers, a syncmer scheme selects *k*-mers from a predetermined subset of all *k*-mers and thus may not select a *k*-mer from every window for a given *w*.

#### Parameterized syncmers

We are now ready to introduce the key new concept of this study. A *parameterized syncmer scheme* (PSS) with parameters *s*, *k*, *o* and *x*_1_, …, *x*_*n*_ where 0 < *x*_1_ < … < *x*_*n*−1_ < *x*_*n*_ ≤ *k* − *s* + 1 selects a *k*-mer if the minimum *s*-mer of that *k*-mer appears at one of the positions *x*_*i*_ in the *k*-mer. Formally:

$$\begin{array}{c} S_{k,s,o,\{x_{1},\ldots ,x_{n}\}}\left(S\right)=\{i|M_{s,k-s+1,o}\left(S\left[i,i+k-1\right]\right)\in \left\{x_{1},\ldots ,x_{n}\right\}\} \end{array}$$ (2)

As *o* is fixed we will drop it from the notation where possible. An example of a PSS is shown in Fig 1B. For convenience, we will denote the PSS with parameters *x*_1_, …, *x*_*n*_ as $S_{k,s}\left(x_{1},\ldots ,x_{n}\right)$, and will drop *k* and *s* from the notation where they are not needed. Under these definitions, the open and closed syncmer schemes are $S_{k,s}\left(1\right)$ and $S_{k,s}\left(1,k-s+1\right)$, respectively.

#### Downsampled and windowed schemes

In some situations we wish to cull the selected *k*-mers or fill in sequence segments where none was selected. Given a uniformly random hash function *h* : Σ^*k*^ → [0, *H*], for a given string *S*, *downsampling* selects syncmers only from the set of |Σ|^*k*^/*δ k*-mers with the lowest hash values. We call *δ* the *downsampling rate*. *Windowed PSS* fill in gaps using a minimizer scheme, thus providing a window guarantee. See Section A in S1 Text for formal definitions of these sets.

#### Canonical *k*-mers

In implementations of PSS we will actually use canonical *k*-mes, defined as follows. Denote the reverse complement of *x* by $\bar{x}$. For a given *k*-mer order, *o*, the *canonical form* of a *k*-mer *x*, denoted by *Canonical*_*o*_(*x*), is the smaller of *x* and $\bar{x}$. For example, under the lexicographic order, *Canonical*_*lex*_(*CGGT*) = *ACCG*. Our theoretical analysis will focus only on forward (non-canonical) *k*-mers.

### Properties and evaluation criteria of schemes

We define some metrics for evaluating the performance of selection schemes.

#### Density and compression

The *density* of a scheme [12] is the expected fraction of positions selected by the scheme in an infinitely long random sequence: d(f)=E[|f(S)|/|S|] as |*S*|→∞. The *compression rate* [7] is defined as *c*(*f*) = 1/*d*(*f*), i.e. the factor by which the sequence *S* is “compressed” by representing it using only the set of selected *k*-mers.

#### Conservation

Conservation [9] is the expected fraction of positions covered by a selected *k*-mer in sequence *S* that will also be covered by the same selected *k*-mer in the mutated sequence *S*′ where *S*′ is generated by iid base mutations with rate *θ*. Define the set of positions covered by the same selected *k*-mer in both sequences

$$\begin{array}{cc} B_{S}\left(f,\theta ,k\right) & =\{i\;|\;\exists j\in \{i-k+1,i-k+2,\ldots ,i\}\\ & s.t.\;j\in f\left(S\right)\cap f\left(S^{\prime }\right)\wedge S\left[j,j+k-1\right]=S^{\prime }\left[j,j+k-1\right]\} \end{array}$$

Then the *conservation* of the scheme is defined as $Cons\left(f,\theta ,k\right)=E_{S}[|B_{S}\left(f,\theta ,k\right)|/\left|S\right|]$.

#### Spread and distance distribution

One key feature of a scheme is the distance between selected positions and the frequency with which selected positions appear close together or far apart. Shaw and Yu [9] studied the probability distribution of selecting *at least one* position in a window of length *α*. We refer to the vector *P*(*f*, *α*) of these probabilities as the *spread*.

We define the *distance distribution* of *consecutive* selected positions: *Pr*(*f*, *n*) is the probability that position *i* + *n* is the next selected position given that position *i* is selected.

#### *pN* metric

The *pN* metric (*N* ∈ [0, 100]) is the *Nth* percentile of the distance distribution, i.e., it is the length *l* for which *N*% of the distances between consecutive selected positions are of length ≤ *l*.

#### *ℓ* and *ℓ*_2_ metrics

A *gap* is a nonempty stretch of sequence between two consecutive selected *k*-mers. Gaps are uncovered by the scheme. Let the lengths of the gaps generated by a scheme on the sequence *S* be *l*_1_, *l*_2_, …. We define $\ell =\frac{1}{\left|S\right|}\sum_{i}l_{i}$ and $\ell_{2}=\sqrt{\frac{1}{\left|S\right|}\sum_{i}l_{i}^{2}}$. Note that the expected value of *ℓ* is 1 − *conservation*.

While these metrics are defined in expectation for given sequence and mutation models and the selection scheme, we also use the analogously defined empirical values measured on a specific sequence. The metrics may also be considered on the positions selected by a scheme in a reference, or only on the selected positions that are *conserved* after mutation or sequencing error. We refer to the latter using the subscript *mut*, for example, *ℓ*_2,*mut*_ is defined analogously to *ℓ*_2_ except the gaps are between consecutive selected *k*-mers that are conserved after mutation.

### Choosing an appropriate metric to compare schemes

**Fig 2:**
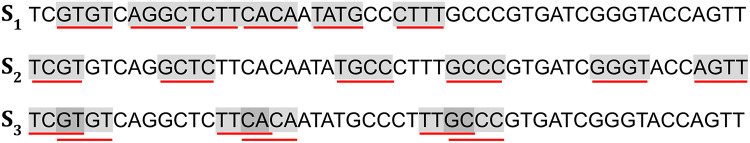
*ℓ* vs. *ℓ*_2_ metric. The selected positions of three different selection schemes S_1_, S_2_ and S_3_ on the same sequence. Selected *k*-mers are highlighted and underlined. All schemes have the same number of selected *k*-mers, but the metrics are different. **S**_**1**_: *ℓ* = 0.529, *ℓ*_2_ = 2.974. **S**_**2**_: *ℓ* = 0.529, *ℓ*_2_ = 1.81. **S**_**3**_: *ℓ* = 0.647, *ℓ*_2_ = 2.808. While S_1_ and S_2_ have the same *ℓ* value, the *k*-mers selected by S_2_ are more evenly spread and thus S_2_ has much lower *ℓ*_2_. Some of the *k*-mers selected by S_3_ overlap, resulting in a higher *ℓ* value than the other schemes. However, because the gaps between covered bases are more evenly spread, the *ℓ*_2_ value is lower than that of S_1_. Intuitively, it will be easier to map reads using seeds selected by S_3_ than S_1_ despite the higher *ℓ* value, suggesting that *ℓ*_2_ is a more appropriate metric.

While Edgar shows convincingly that conservation is a more appropriate metric for comparing selection schemes than density, we argue that *ℓ*_2,*mut*_ contains additional important information for the purpose of mapping. Specifically, observe that, for given mutation rate *θ*, *k*, and selection scheme *f*, we have $E[\ell_{mut,\theta ,f,k}]=1-Cons\left(\theta ,f,k\right)$. While *ℓ* (and conservation) counts the number of bases that are not covered by conserved selected *k*-mers, it treats all gap lengths equally. In contrast, *ℓ*_2,*mut*_ penalizes a few large gaps more than many smaller gaps with the same total length. See the example in Fig 2. When the selected *k*-mers are used as seeds for mapping, it is important to avoid large gaps, in order to enable read mapping across gaps. Thus, while *ℓ* and *ℓ*_2_ are correlated, *ℓ*_2_ provides additional information on how the selection scheme may affect mapping performance, and we use it throughout to select the scheme for given values of *k*, mutation, and compression in our syncmer-based mappers.

### Analysis of syncmer schemes—Prior work

Edgar recently defined syncmers as an alternative to minimizers and other selection schemes with the goal of improving conservation rather than density, arguing that density is often dictated by the application and system constraints [7]. He introduced open and closed syncmers and their rotated variants. Analyses of syncmer densities, window guarantees, and distributions were provided for open, closed, and downsampled syncmers. Note that the window guarantee for closed syncmers is for a fixed *w* = *k* − *s* for *k*-mer and *s*-minimizer lengths *k* and *s* rather than for any given *w*.

Shaw and Yu greatly extended the framework for theoretical analysis of syncmers [9]. They defined the spread and conservation of a scheme. The two are connected through the number of unmutated *k*-mers overlapping a given position, *α*(*θ*, *k*), for a given mutation rate, *θ*. Letting *P*(*f*) = [*P*(*f*, 1), *P*(*f*, 2), …*P*(*f*, *k*)] be the spread, and *P*(*α*(*θ*, *k*)) = [*P*(*α*(*θ*, *k*) = 1), *P*(*α*(*θ*, *k*) = 2), …, *P*(*α*(*θ*, *k*) = *k*)], then *Cons*(*f*, *θ*, *k*) = *P*(*f*) ⋅ *P*(*α*(*θ*, *k*)). Note that there is a closed form expression for calculating *P*(*α*(*θ*, *k*) = *α*)), and that *P*(*f*, 1) = *d*(*f*). Their theoretical framework allowed Shaw and Yu to obtain expressions for the spread (and therefore conservation) of open and closed syncmers and other selection schemes.

## Methods

In this section we first outline the main results of our theoretical analysis of PSS. These results provide guidance for choosing PSS parameters in practice. We then describe how we modify mappers to utilize them. Due to space constraints the full derivations and analysis are deferred to Section B in S1 Text. Raw data for results presented in this and subsequent sections are available in S1 Data.

### Recursive expressions for conservation of PSS

Shaw and Yu [9] obtained expressions for open and closed syncmer conservation as a function of spread. Here we present a general recursive expression for the spread of any PSS, including with downsampling. These expressions allow for the calculation of the conservation of any PSS. The full derivation of the general expression is presented in Section B.1 of S1 Text while here we present only the final expression itself.

**Fig 3:**
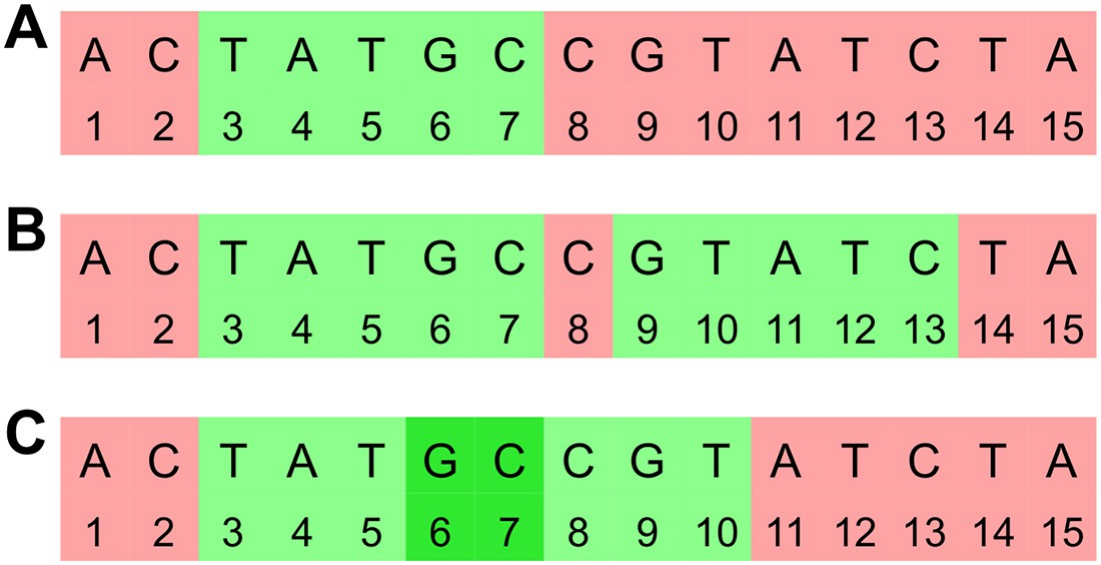
Illustration of *s*-minimizers generating syncmers. A window of *α* = 5 consecutive 11-mers. **A**: When *s* = 5 and *t* = 3, then the *s*-minimizer of the entire window generates a syncmer when its starting index is in the green region. If the *s*-minimizer is in one of the red regions then a syncmer may be generated by the *s*-minimizer of the remaining part of the window. For a two parameter scheme the *s*-minimizer creates two syncmer generating regions that may be disjoint (**B**) if *s* > *t*_2_ − *t*_1_ or overlapping (**C**) if *s* < *t*_2_ − *t*_1_. In this example, *t*_1_ = 3 and *t*_2_ = 9 in **B** and *t*_2_ = 6 in **C**.

Consider a window of *α* consecutive *k*-mers. We assume random sequence (i.e., made up of iid bases) throughout. Let *s*_*β*_ be the *s*-minimizer in the *α*-window, at position *β*. Then if *t* is a parameter of the syncmer scheme, *s*_*β*_ generates a syncmer if it is not in the first *t* − 1 or last *k* − *t* positions in the *α*-window. If *β* is not in a position where it generates a syncmer, we recursively check to the left or right of *β* to see if a syncmer is generated by the *s*-minimizer of that region. See Fig 3 for an example.

For a PSS *f* with *k*-mer length *k*, *s*-minimizer length *s*, and downsampling rate *δ*, let *P*(*α*) be the probability of selecting at least one syncmer in a window of *α* adjacent *k*-mers. We assume a uniformly random hash over the *s*-mers, and condition on the position, *β*, of the *s*-minimizer in the *α*-window. For each *β* we sum over two cases: 1) *β* generates at least one syncmer that is not lost due to downsampling, 2) *β* does not generate a syncmer, or all are lost due to downsampling, in which case a syncmer may be generated by the part of the window to the left or right, resulting in a recursive expression. Let *P*_*R*_ = *P*(*α* − *β*) and *P*_*L*_ = *P*(*β* − *k* + *s* − 1). Then we have:

$$\begin{array}{cc} P(\alpha ) & =p_{\beta }\sum_{\beta }P\left(\alpha |\beta \right)\\ & \approx \frac{1}{k+\alpha -s}\cdot \sum_{\beta =1}^{k+\alpha -s}[(1-{\left(1-\frac{1}{\delta }\right)}^{count(\beta )})+{\left(1-\frac{1}{\delta }\right)}^{count(\beta )}(P_{R}+P_{L}-P_{R}\cdot P_{L})] \end{array}$$

The probability of any of the *k* + *α* − *s* starting positions being the *s*-minimizer is denoted as *p*_*β*_ and assumed to be uniform. This assumption starts to break down when the *s*-minimizer is not unique, thus we note that the probability is approximate. *count*(*β*) represents the number of syncmers generated by the *s*-minimizer *s*_*β*_. For example, *count*(*β*) = 0 in the red region of Fig 3 and *count*(*β*) = 2 in the overlapped region when *β* = 6 or 7 in Fig 3C. Note we define *P*(*α*) = 0 when *α* ≤ 0.

### Calculating *ℓ*_2,*mut*_

We compute *ℓ*_2,*mut*_ using the distance distribution. Let *D*(*α*) represent the probability that the distance between two adjacent syncmer positions is *α* − 1, and *D*_*mut*_(*α*) be the same under mutation, then the expressions for *ℓ*_*mut*_ and *ℓ*_2,*mut*_ can be written as:

$$\begin{array}{c} \ell_{mut}=\sum_{x=k+1}^{\infty }\left(x-k\right)\cdot D_{mut}\left(x+1\right) \end{array}$$ (3)

$$\begin{array}{c} \ell_{2,mut}=\sqrt{\sum_{x=k+1}^{\infty }{\left(x-k\right)}^{2}\cdot D_{mut}\left(x+1\right)} \end{array}$$ (4)

To calculate *D*(*α*) we define the new quantity *F*(*α*) denoting the probability that *only* the first or *only* the last *k*-mer in a window of *α* *k*-mers is a syncmer, respectively. We refer to these *k*-mers as *K*_1_ and *K*_*α*_ respectively. Note that for *P*(*α*) defined as above, 1 − *P*(*α*) gives the probability that *no k*-mer in an *α*-window is a syncmer.

We compute *F*(*α*) by conditioning on *β* as before. For simplicity we divide the sum over *β* into cases based on the syncmers that are generated by *s*_*β*_. With some abuse of notation, we let *K*_*i*_ represent the event that *s*_*β*_ generates *K*_*i*_ as a syncmer.

$$\begin{array}{cc} F\left(\alpha \right)\approx \sum_{\beta =1}^{k+\alpha -s}\frac{1}{k+\alpha -s} & \times \{\begin{array}{cc} \frac{1}{\delta }.{\left(1-\frac{1}{\delta }\right)}^{count(\beta )-1}\cdot \left(1-P\left(\alpha -\beta \right)\right) & K_{1}\\ {\left(1-\frac{1}{\delta }\right)}^{count(\beta )}\cdot F\left(\beta -k+s-1\right)\cdot \left(1-P\left(\alpha -\beta \right)\right) & \text{otherwise} \end{array} \end{array}$$

In the first case we have the probability that *K*_1_ is not downsampled, any other syncmer generated by *s*_*β*_ is downsampled, and there are no other syncmers generated to the right of *β*. In the second case we have the probability that any syncmers generated by *s*_*β*_ are downsampled, no syncmers are generated to the right of *β*, and the recursive computation of the probability that the *s*-minimizer of the segment to the left of *β* generates a syncmer at *K*_1_.

Similarly, *D*(*α*) is the probability that in a window of *α k*-mers *only* the first *and* last *k*-mers are syncmers. Then

$$\begin{array}{cc} D(\alpha )\approx & \sum_{\beta =1}^{k+\alpha -s}\frac{1}{k+\alpha -s}\times \{\begin{array}{cc} {\left(\frac{1}{\delta }\right)}^{2}\cdot {\left(1-\frac{1}{\delta }\right)}^{count(\beta )-2} & K_{1},K_{\alpha }\\ \frac{1}{\delta }\cdot {\left(1-\frac{1}{\delta }\right)}^{count(\beta )-1}\cdot F\left(\alpha -\beta \right) & K_{1},\neg K_{\alpha }\\ \frac{1}{\delta }\cdot {\left(1-\frac{1}{\delta }\right)}^{count(\beta )-1}\cdot F\left(\beta -k+s-1\right) & K_{\alpha },\neg K_{1}\\ {\left(1-\frac{1}{\delta }\right)}^{count(\beta )}\cdot F\left(\beta -k+s-1\right)\cdot F\left(\alpha -\beta \right) & \text{otherwise} \end{array} \end{array}$$

To compute *ℓ*_2,*mut*_ we need the analogous expressions *F*_*mut*_(*α*) and *D*_*mut*_(*α*). The expressions for these values are more involved, and left to Section B.3 of S1 Text. For a given mutation rate and compression, we compute the theoretical *ℓ*_2,*mut*_ of all PSS according to these expressions. We can then select the PSS with parameters yielding the lowest *ℓ*_2,*mut*_.

Because of the recursive nature of the theoretical expressions, their computation even to a fixed accuracy is time consuming. In practice, simulating a very long sequence, selecting syncmers, and simulating mutations to determine this metric empirically is less time consuming. Our tests show that the theoretical and empirical results are very close. For example, for $S_{15,5,lex}\left(i,j\right)$ for 1 ≤ *i* < *j* ≤ 11 and 15% mutations, the average difference was 0.26%. (See Table A in S1 Text and Table C in S1 Data). We used this simulation method to compute *ℓ*_2,*mut*_ for *k* = 11, 13, 15, 17 and 19, mutation rates 0.05, 0.15 and 0.25, and all 2- and 3-parameter schemes. The results are presented in Table B of S1 Data (note that for 1-parameter schemes the best *ℓ*_2_ and *ℓ* are the same, and thus already known from [9]). *ℓ*_2,*mut*_ values computed using the theoretical expressions for some parameter combinations are available in Table C of S1 Data.

### Achieving the target compression

A simple extension of the expression for compression of open and closed syncmers yields that the compression of an *n*-parameter PSS is $\approx \frac{k-s+1}{n}$, where we assume that *s* is long enough relative to *k* so that the *s*-minimizer is likely to be unique. Table D of S1 Data contains the *ℓ*_2,*mut*_ values for schemes that achieve the same compression either by using more parameters or by downsampling. The table shows that it is preferable to achieve a specific compression with minimal downsampling. For example, the *ℓ*_2_ of the best 2-parameter scheme with a downsampling rate of 2 is an order of magnitude worse than that of the best 1-parameter scheme that has the same compression without downsampling. Thus, to choose the PSS with best *ℓ*_2,*mut*_ for a given target compression, we can choose one with parameters that yield the compression closest to, but below, the desired compression and then downsample to reach the desired compression.

Note that while for 1-parameter PSS the scheme with best conservation (and *ℓ*_2_) always has its *s*-minimizer in the middle position as shown by Shaw and Yu [9], for multi-parameter PSS, which scheme has best *ℓ*_2,*mut*_ may change depending on mutation rate and compression, and there is no scheme that has lowest *ℓ*_2,*mut*_ in every setting. Table B in S1 Data is used to select the scheme with lowest *ℓ*_2,*mut*_ for a given setting.

### Implementing PSS in mappers

We modified the code of minimap2 (v2.22-r1105-dirty) and Winnowmap2 (v2.03) to select our syncmer variants as seeds instead of minimizers. The code for our new syncmer-based mappers is available from https://github.com/Shamir-Lab/syncmer_mapping.

The implementation of the syncmer schemes defined in Definitions and background is straightforward. Sequences are scanned from left to right, the canonical *k*-mer (under random hash *h*_1_) at each position is identified, and the index of the minimum *s*-mer under random hash *h*_2_ is determined, and compared against the list of allowed positions of the PSS. Note that, like the minimizers in the original mappers, the PSS implementation uses canonical encoding of the *k*-mers. This means that the theoretical analysis above does not hold exactly for these schemes, however, in practice, the overall trend holds as we will show in the Results section.

For downsampled schemes, syncmers are selected if their hash value normalized between 0 and 1 is below 1/*δ* where *δ* is the downsampling rate. Note that *h*_2_, a different hash function than *h*_1_ must be used to ensure random downsampling. Windowed schemes are integrated into the minimizer selection scheme of the mappers except that syncmers are selected in each window first. If no syncmer is present, then the minimizer is selected.

Pseudocode describing these implementations is presented in Algorithm 1 for regular PSS and in Algorithm A in Section C of S1 Text for windowed PSS. Additional implementation and optimization details are presented in Section D of S1 Text.

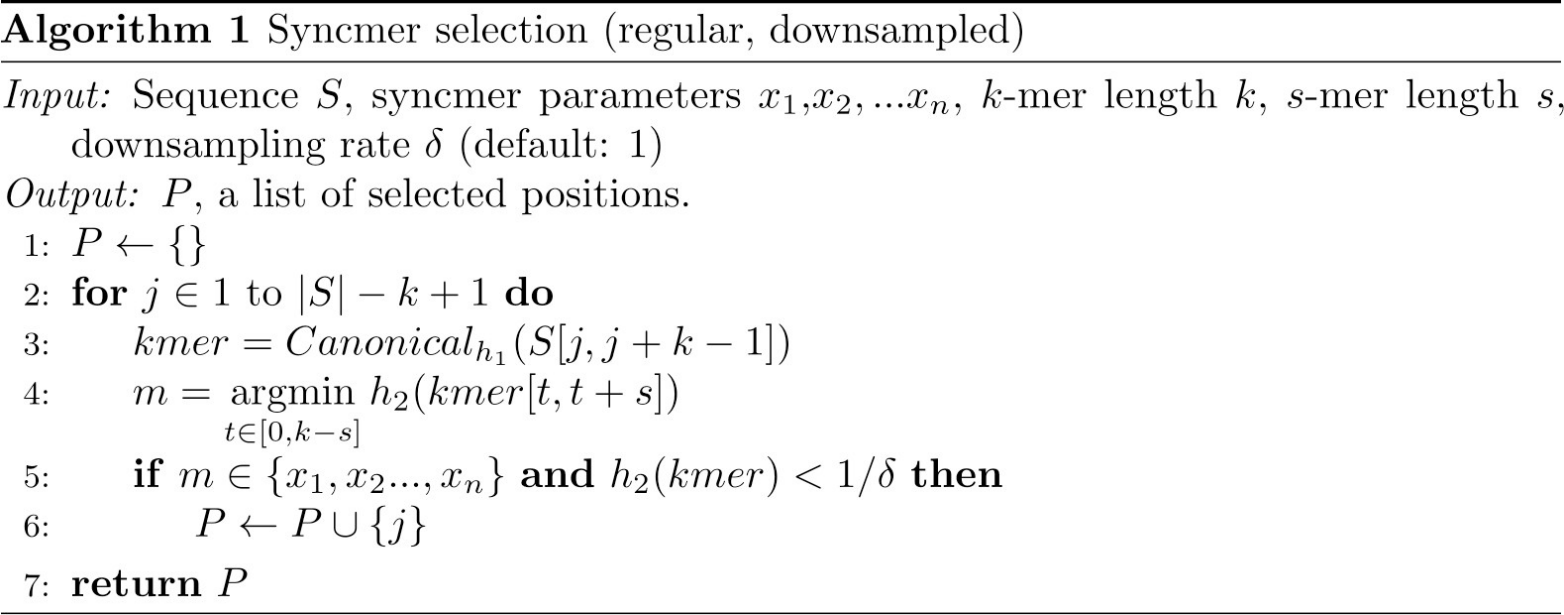


## Results

We first evaluate different PSS on real genomes to demonstrate their properties compared to the theoretical analysis presented above. We then compare PSS-based mapping to the original minimizer-based versions of minimap2 and Winnowmap2 on simulated and real read data, using the theoretical analysis to select schemes with best *ℓ*_2,*mut*_. We used canonical *k*-mers in our experiments. The reasons for this choice are explained in subsection “The impact of using canonical *k*-mers” below. We show that, under realistic mutation rates, these schemes perform not only better than minimizers, but also better than closed syncmers, which do not have optimal *ℓ*_2,*mut*_. Parameters and command lines used for all tools are shown in Section E of S1 Text.

The reference sequences used for these experiments were: human genome GRCh38.p13 [13], human chromosome X from CHM13 (v1.0) [14], *E. coli* K12 [15], and a set of microbial genomes that we will call BAC, containing assemblies of 15 microbes for which PacBio long-read data is available [16] (three of the microbes were used in [9], see Section G in S1 Text for more details). Information about the sequences is presented in Table 1.

**Table 1:** Reference genomes. Basic information about the reference genomes used in our experiments. # scaffolds is the number of individual sequences present in the reference genome fasta file and can include unplaced scaffolds, alternates, etc. Length is the total length (in nt) of all of the scaffolds together, excluding ambiguous bases.

Dataset	Source	Species	# scaffolds	Length
GRCh	GRCh38 [13]	Human	639	3.111G
CHM13X	CHM13 chrX [14]	Human	1	154.3M
BAC	PacBio [16]	Microbial	24	59.1M
ECK12	GCF_000005845.2 [15]	*E. coli K-12*	1	4.6M

We simulated PacBio and ONT reads from the human genome and from BAC with a depth of 10 using PBSIM [17] and NanoSim [18]. Details of simulation parameters are found in Section F in S1 Text.

For tests on real read datasets we selected a random set of 10K ONT reads of the NA12878 cell line with read length capped at 10kb (SRA accession ERR3279003), and 1K PacBio reads for each of the BAC microbes [16]. Details are available in Table 2.

**Table 2:** Reads information. The long-read datasets used in our experiments. Source names are from Table 1 where relevant. PB = PacBio, ONT = Oxford Nanopore Technologies.

Dataset	Source	Read type	# reads	Mean length (std)
pbsim_x	CHM13X	PB simulated	173891	8871.1 (5570.1)
pbsim_bac	BAC	PB simulated	66428	8894.2 (5617.4)
ns_chm13	CHM13	ONT simulated	1000	8722.8 (7030.7)
pb_bac	BAC	PB real	15000	9488.3 (5207.2)
ont_na12878	ERR3279003	ONT real	10000	7131.6 (2348.5)

### Performance of parameterized syncmer schemes

Our theoretical analysis of PSS properties above relies on a number of assumptions. Specifically, it assumes uniform iid sequences and mutations, allows substitutions only, and treats the sequence as a single forward strand. We therefore examined the properties of PSS on real genomes where these assumptions do not necessarily apply, and compared them to minimizer schemes.

We used *k* = 15 and selected the PSS with best *ℓ*_2,*mut*_ (“optimal PSS”) with theoretical compression 5.5 and 10 (S(3,9) and S(6), respectively). The default minimizer scheme of minimap2 uses *k* = 15 and *w* = 10 yielding the theoretical compression of 5.5. A theoretical compression of 10 is achieved by minimap2 with *k* = 15 and *w* = 19. For compression 5.5 we also included in the comparison closed syncmers (i.e. S(1,11)) and a PSS that should perform poorly according to the theoretical analysis (“bad PSS”, S(1,2)). We compared the schemes on both unmutated sequences and on sequences with iid substitutions simulated at a rate of 15%. Since conservation is defined for index-preserving mutations, indels were not simulated (sequencing errors were included in all subsequent simulations in the following sections).

We tested the schemes on the ECK12 and CHM13X sequences, with and without mutations. On *unmutated* reference sequences, minimizers outperformed PSS, with much lower *ℓ*_2_ and *p*100 values for schemes with the same compression (Table B in Section H of S1 Text). The theoretically best PSS outperformed the closed syncmer scheme and the “bad PSS”. In contrast, under mutation, the advantage of syncmers is clear (Table 3): PSS had better performance in all metrics, with the theoretically best PSS performing better than minimizers and closed syncmers. This holds true even for relatively low mutation rates of less than 5% (Table C in S1 Text). While PSS had significantly more conserved positions than minimizers, the “bad PSS” S(1,2) had a worse distribution of selected positions and thus poorer *ℓ* and *ℓ*_2_ than minimizers.

**Table 3:** Performance metrics of minimizer and syncmer schemes on real sequences with simulated mutations. Substitutions were introduced in the references at a rate of 15%. The values shown are for the conserved selected *k*-mers. # conserved is the number of *k*-mers selected by a scheme that were conserved under mutation. Best performance is shown in bold. “Optimal PSS” refers to the PSS with the lowest theoretical *ℓ*_2,*θ*_ (Table SD2) for *θ* = 0.15.

Dataset	Scheme	Description	Compression	*ℓ*	*ℓ*_2_	*p*90	*p*100	# conserved
ECK12	$M_{15,10}$	minimap minimizer	76.65	0.86	13.77	211	1045	60,557
	$S_{15,5}\left(3,9\right)$	optimal PSS	62.91	**0.84**	**12.97**	**182**	**941**	73,779
	$S_{15,5}\left(1,11\right)$	closed syncmer	63.37	0.85	13.42	188	1277	73,245
	$S_{15,5}\left(1,2\right)$	“bad PSS”	63.23	0.87	14.19	195	1443	73,413
	$M_{15,19}$	minimap minimizer	154.13	0.91	17.85	378	1981	30,115
	$S_{15,6}\left(6\right)$	optimal PSS	116.04	**0.90**	**16.18**	**303**	**1542**	40,001
CHM13X	$M_{15,10}$	minimap minimizer	54.29	0.81	11.70	152	1219	2,841,498
	$S_{15,5}\left(3,9\right)$	optimal PSS	45.65	**0.80**	**11.12**	**132**	**1193**	3,379,361
	$S_{15,5}\left(1,11\right)$	closed syncmer	44.71	0.81	11.46	134	1248	3,450,241
	$S_{15,5}\left(1,2\right)$	“bad PSS”	43.87	0.82	12.07	137	1387	3,515,931
	$M_{15,19}$	minimap minimizer	107.91	0.88	15.12	270	1946	1,429,555
	$S_{15,6}\left(6\right)$	optimal PSS	83.20	**0.86**	**13.83**	**219**	**1927**	1,854,097

### The fraction of unmapped reads

We mapped reads using minimap2 and Winnowmap2 with $M_{15,10}$ (low compression), $M_{15,50}$ (medium), and $M_{15,100}$ (high) on four datasets. For each dataset, syncmer-minimap and syncmer-winnowmap parameters yielding the best theoretical *ℓ*_2,*mut*_ for the same compression achieved by minimap2 were selected. This resulted in $S_{15,5}\left(3,9\right)$ matching the low compression, and $S_{15,4}\left(6\right)$ matching the medium and high compression. The downsampling rate was manually selected to match the real compression of the corresponding minimizer scheme as closely as possible. The exact compression and downsampling rates are given in Table E in S1 Data. For PacBio reads homopolymer compression was used by all mappers. Note that for ONT reads Winnowmap2 uses SV mode.

**Fig 4:**
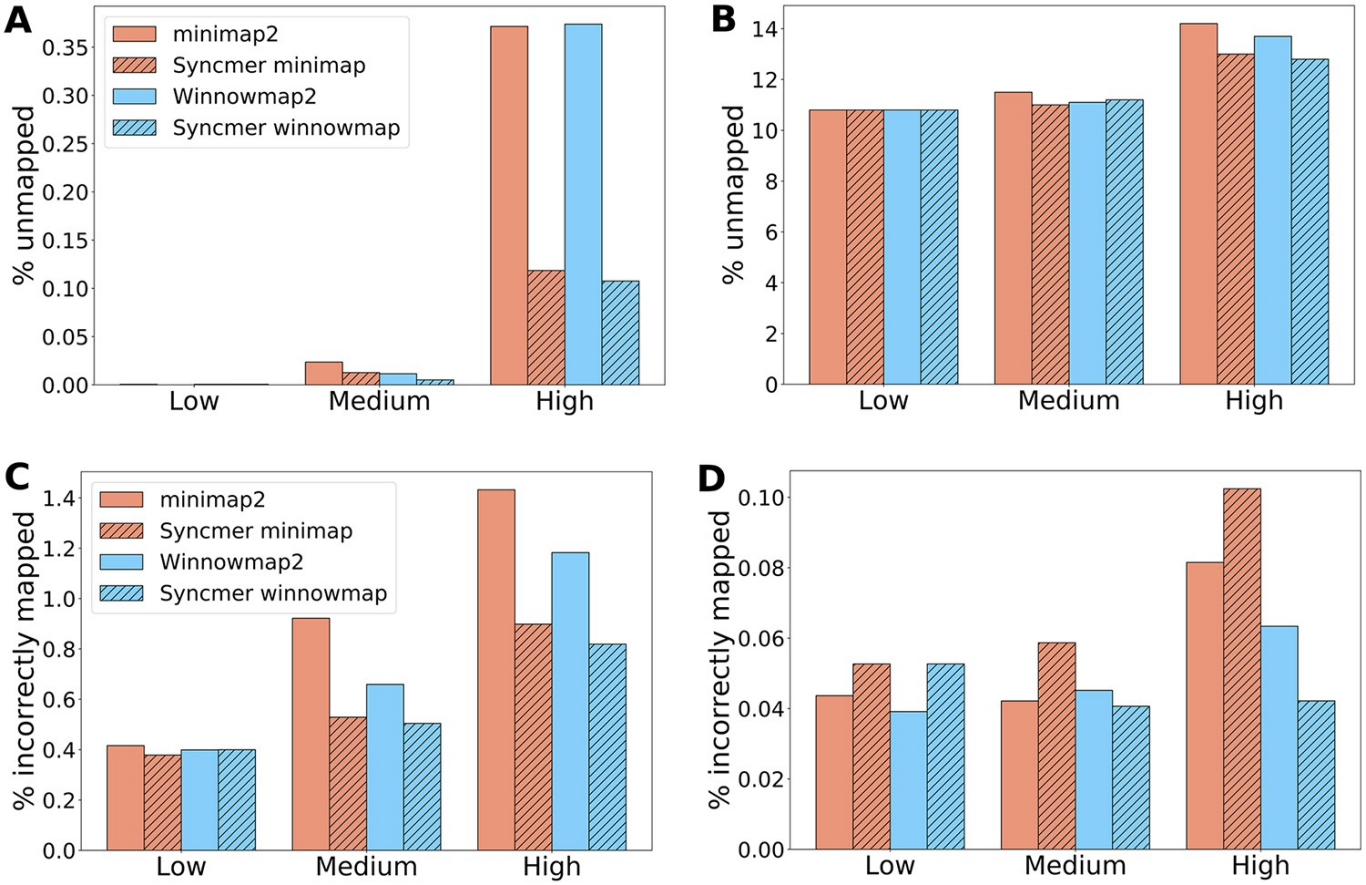
The percentage of unmapped and incorrectly mapped reads—simulated data. **Top**: Percent unmapped for low, medium and high compression. **(A)** PacBio reads simulated from the CHM13X sequence mapped against ChrX sequences from GRCh38; **(B)** 1000 ONT reads simulated from CHM13 mapped against GRCh38. **Bottom**: The percentage of incorrectly mapped reads for low, medium and high compression. **(C)** PacBio reads simulated from the CHM13 ChrX sequence mapped against CHM13X; **(D)** PacBio reads simulated from the 15 bacterial species in BAC pooled together and mapped against the union of their references.

Fig 4 (top) shows the percentage of unmapped reads of the mappers for simulated PacBio and ONT reads mapped to the human reference genome. See Fig D in S1 Text. for additional results, including windowed mappers. Syncmer variants performed essentially the same or better than the original mappers in all cases, with the largest advantage at high compression. All mappers did much better on the PacBio reads than on ONT reads, which have a higher proportion of deletions and substitutions. The jump in the fraction of unmapped reads between medium and high compression may indicate that in order to overcome the large fraction of non-conserved seeds, existing mappers need to use a lower compression with many redundant seeds.

**Fig 5:**
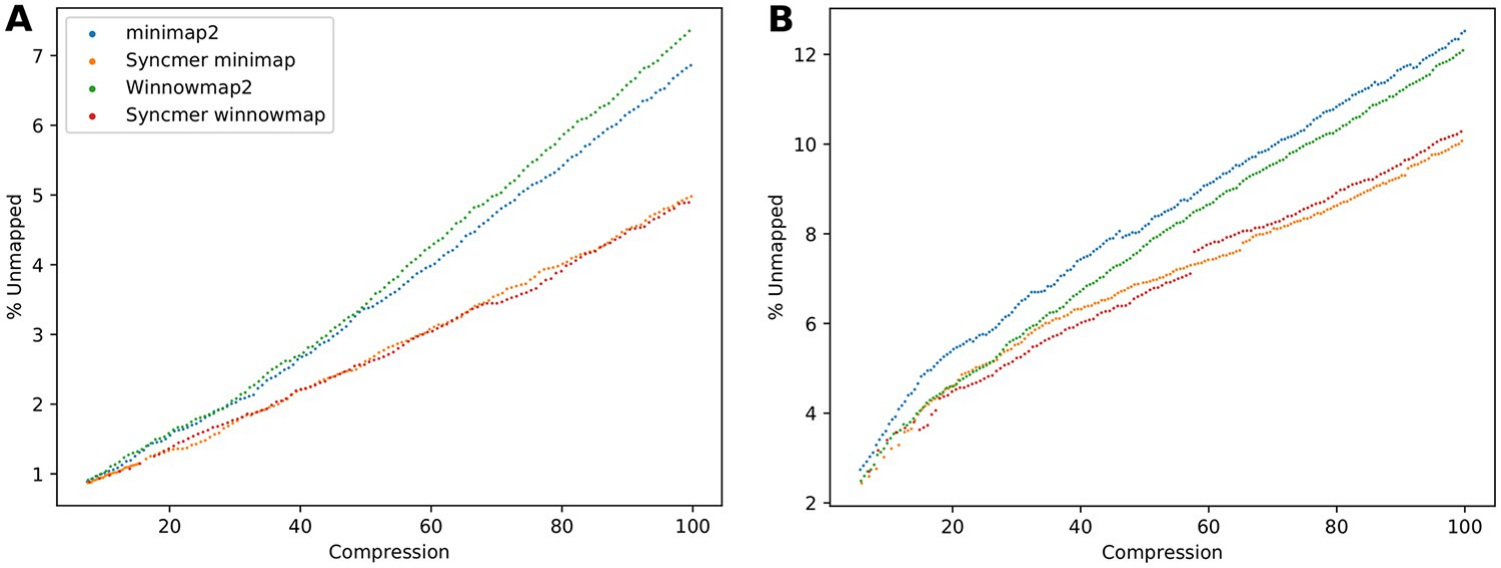
Percentage of unmapped reads—Real datasets. Percentage is shown as a function of compression rate, PSS parameters were chosen to achieve the desired compression with lowest *ℓ*_2,*mut*_. **(A)** Pooled PacBio bacterial reads mapped against BAC. **(B)** ONT human cell-line reads mapped against GRCh38.

We compared the performance of all mappers on real data (Table 2) across a range of compression values. The ONT reads were mapped against the human reference GRCh and the PacBio bacterial reads were mapped against the BAC reference. For the original minimap2 and Winnowmap2 different values of compression were achieved by varying *w*. For the syncmer variants, schemes were selected with the best *ℓ*_2,*mut*_ according to the theoretical analysis and then downsampled, as discussed above (Achieving the target compression). Results are shown in Fig 5. The syncmer variants had consistently higher percentage of mapped reads than the original minimizer-based mappers, with syncmer-winnowmap performing the best across the larger part of the compression range. For high compression, the minimizers had 20–40% more unmapped reads than the syncmers. At low compression rates of 5.5–11, minimizers had 2–15% more unmapped reads than syncmers. Full results and scheme parameters are given in Table F of S1 Data.

To compare the performance of PSS to the original syncmers, we mapped the PacBio bacterial reads against BAC using syncmer-minimap with $S_{15,5}\left(3,9\right)$, the theoretically best 2-parameter scheme, and with closed syncmers (equivalent to $S_{15,5}\left(1,11\right)$) across a range of compression values. The results are shown in Fig E of S1 Text. The PSS selected by our analysis had consistently fewer unmapped reads than closed syncmers. Note that 1-parameter PSS and open syncmers with offsets are equivalent and the best scheme always has its *s*-minimizer in the middle position as discussed above and by Shaw and Yu [9]. In addition, the compression achieved by 3-parameter schemes is lower than necessary to achieve good mapping performance. Thus, the main advantage of PSS over syncmers is for 2-parameter schemes.

### Mapping correctness

We evaluated the mapping correctness for PacBio simulated reads as done in [1] (see Section F in S1 Text for details). The percentage of the *mapped* reads simulated from CHM13X and the BAC genomes that were incorrectly mapped is shown in Fig 4 (bottom). Winnowmap was consistently better than minimap, and the syncmer variants of Winnowmap performed best at medium and high compression. On the BAC genomes (bottom right) syncmer minimap performed worse than the regular minimap, but % incorrectly mapped was very low for all mappers. Note that when mapping rates are different, the percent incorrectly mapped may not be directly comparable.

Although we cannot evaluate the mapping correctness on the real datasets, the mapping quality scores reported by minimap2 can be used to compare the different mappers. On the real datasets, reads mapped by syncmer-minimap but not by minimap2 generally had higher mapping quality than those mapped by minimap2 and not syncmer-minimap. For example, for the human cell line ONT reads, comparing minimap2 with $M_{15,50}$ to syncmer-minimap, the 39 minimap-only reads had average mapping quality 31.4 (median 27), while the 94 syncmer-minimap-only reads had an average quality score of 38.7 (median 42.5). Full results for different compression rates are presented in Table G of S1 Data.

### Impact of sequence identity level

**Fig 6:**
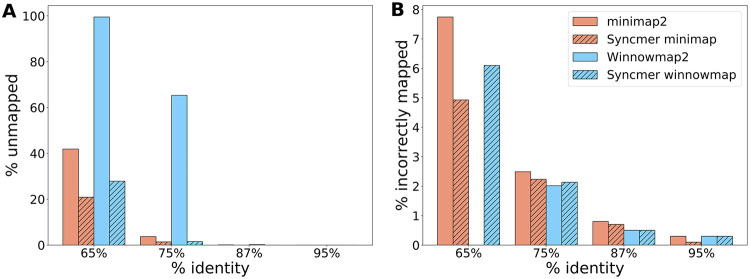
Impact of percent sequence identity on mapping quality. We varied the mutation rate of 1000 PacBio simulated reads from CHM13X. The figures present the % unmapped and incorrectly mapped by each method. **(A)** % unmapped reads. **(B)** % of the mapped reads that were incorrectly mapped.

We examined the impact of the level of identity between the sequenced reads and the reference to which they are aligned. Differences between the sequences can be due to sequencing errors, mutations in the sequenced organism, or differences between sequenced and reference strains. We simulated 1000 PacBio reads from CHM13X at percent identity 65%, 75%, 87% and 95%. The results are shown in Fig 6 and H in S1 Text. For minimap2 and Winnowmap2 we used $M_{15,50}$, and in the syncmer variants we used $S_{15,4}\left(6\right)$ with the other parameters selected as above to match the compression of minimap2.

The syncmer variants outperformed the original tools in terms of fraction of reads mapped, with larger gains as percent identity decreases. All tools performed very well at higher percent identity, indicating that more than enough seeds were selected and conserved to adequately map all reads (and thus perhaps compression could be increased). Winnowmap2 performed noticeably worse at lower percent identity, leaving almost all reads unmapped at 65% identity. Syncmer-minimap outperformed minimap2 on the fraction of correctly mapped reads in all cases. Winnowmap2 correctly mapped a larger fraction of the mapped reads at 75% identity, but mapped only 35% of the reads, compared to ≥ 95% for the other variants. At 95% identity the syncmer variants had fewer incorrectly mapped reads. While very low percent identity may be unrealistic in some cases, these results highlight the impact of the increased conservation of syncmers.

### Performance of windowed syncmer schemes

Windowed schemes combine syncmers and minimizers, complementing the syncmer scheme to provide a window guarantee. Section K of S1 Text presents the results of the experiments on windowed PSS. Although windowed schemes perform better than the unwindowed on some metrics (compare Table F in S1 Text and Table 3), in practice the windowed variants of our syncmer mappers were similar or worse than the variants without windowing for the same compression in most cases (Figs F-I in S1 Text).

### The impact of using canonical *k*-mers

In practice, since canonical *k*-mers are used in the mappers that we modified, we implemented PSS using canonical *k*-mers, in order to enable a fair comparison to them. We did so although the theoretical analysis used to select the PSS was developed for single stranded sequences. The selection of canonical *k*-mers can be thought of as selection of forward *k*-mers on each strand, followed by culling of the non-canonical *k*-mers from the union set. The distribution of distances between selected positions on each strand are the same, assuming random DNA. Therefore, we hypothesize that PSS with good theoretical properties in the forward strand analysis will preserve these properties in this selection process.

To test the effect of using canonical *k*-mers, we recomputed the performance metrics shown in Table 3 using only forward (non-canonical) *k*-mers. Table D in S1 Text shows that there are only minor differences in the ranking of schemes when using forward-only and canonical *k*-mers. In addition, *ℓ*_2,*mut*_ values for non-canonical *k*-mers were computed by simulation for some values of *k*, *s*, and mutation rate, and are reported in Table H of S1 Data. Again, there were only minor changes in the rankings of schemes, as expected (Table I in S1 Data).

We also wished to test the impact of using canonical *k*-mers on the distance distribution between selected positions. Fig B in S1 Text shows the distance distributions for syncmers selected only using forward strand *k*-mers and using canonical *k*-mers. We conclude that while the theory is limited to single-stranded sequences it shows trends that hold for canonical *k*-mers. Further details can be found in Section I of S1 Text.

**Fig 7:**
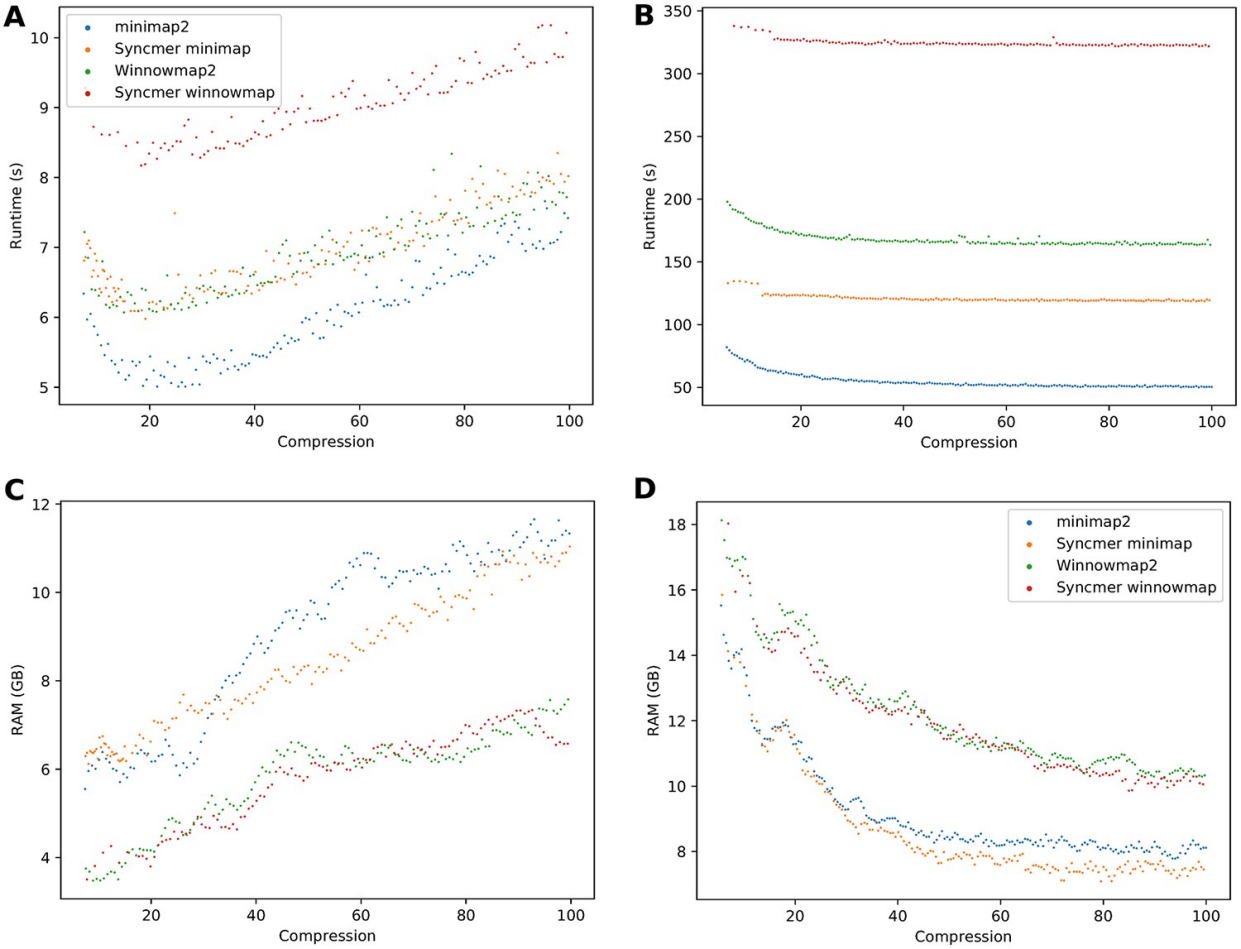
Memory usage and runtime vs. compression—Real data. **(A,B)** Runtime in seconds to index the reference and map reads by each method. **(C,D)** Peak RAM usage in GB to index the reference and map reads. **(A)** and **(C)** are on PacBio bacterial reads. **(B)** and **(D)** are for ONT human cell-line reads.

Finally, as another comparison of the possible effect on mapping to minimizer-based mapping we artificially disabled seed selection from the reverse strand (i.e. allowing only forward *k*-mers to be selected) in the indexing and mapping stages of minimap2 and the syncmer variant. Results are shown in Fig C in S1 Text and should be compared to Figs 5 and 7. Although this drastically reduces mapping performance, we still observe the same mapping performance improvement gained by using syncmers.

### Runtime and memory

We compared the runtime and memory usage of the six tested mappers on a number of datasets. All experiments were performed on a 44-core, 2.2 GHz server with 792 GB of RAM, using 50 threads. Peak RSS (in GB) and real time (in seconds) as measured by the tools are reported.

Table 4 compares the separate performance of indexing and mapping on simulated PacBio and ONT reads from bacteria and human. Winnowmap was not compared as it does not allow for separate indexing and mapping, a disadvantage when many read sets will be mapped to the same reference. For syncmer-minimap the same parameters matched to the minimizers as above were used. At low compression minimap2 had better runtimes for both indexing and mapping, and memory usage was similar between the tools. At high compression syncmer-minimap had longer indexing time but lower mapping time and required less than half the memory. This is in addition to having only 1/3 as many unmapped reads (Fig D(A) in S1 Text).

**Table 4:** Runtime and memory. Time (in seconds) and RAM (in GB) needed to index the reference and map the simulated reads by each of the tools. The second and third dataset use the same reference. Syncmer variant parameters were selected to match the minimap2 compression rates as above.

Task	Method	Scheme	Index time	Index mem	Map time	Map mem
bacterial reads vs BAC	minimap2	$M_{15,10}$	**3.29**	0.32	**11.10**	3.14
	Syncmer minimap	$S_{15,5}\left(3,9\right)$	3.81	**0.31**	11.63	**2.93**
ChrX reads vs CHM13X	minimap2	$M_{15,10}$	**7.96**	1.01	**65.29**	**5.35**
	Syncmer minimap	$S_{15,5}\left(3,9\right)$	9.52	**0.99**	141.05	6.73
	minimap2	$M_{15,100}$	**4.4**	0.45	59.05	16.06
	Syncmer minimap	$S_{15,4}\left(6\right)$	7.12	0.45	**47.83**	**7.56**
bacterial reads vs CHM13X	minimap2	$M_{15,10}$	As above	As above	**25.61**	**8.26**
	Syncmer minimap	$S_{15,5}\left(3,9\right)$			46.59	9.28

We also compared the runtime and memory of all the runs for different compression rates shown in Fig 5. Results are shown in Fig 7. Note that the results here are for indexing and mapping together. minimap2 was consistently the fastest, followed by syncmer-minimap, which took 50–100% longer. Interestingly, the two datasets show opposite trends in memory usage (Fig 7C and 7D). This is because the bacterial reference genomes are relatively short, and thus the memory bottleneck is in the mapping stage, while for the human reference genome the memory bottleneck is in the indexing stage. Increasing compression lowers index size but results in longer alignments between seeds, requiring more memory in the mapping phase. Thus, when indexing is the bottleneck, increasing compression reduces memory, while when mapping is the bottleneck it increases memory. Winnowmap2 and its variants used less memory in the mapping phase while minimap2 and its variants used less memory in the indexing phase. In the case that indexing was the bottleneck, the syncmer variants required lower memory usage than the original mappers across most of the range of compression rates (Fig 7D).

## Discussion

In this study we generalized the notion of syncmers to PSS and derived their theoretical properties. We incorporated PSS into the long-read mappers minimap2 and Winnowmap2. Our syncmer mappers outperformed minimap2 and Winnowmap2, by mapping more reads and correctly mapping a higher fraction of those mapped across a range of different compression values for multiple real and simulated datasets.

As our results show, the advantage of using syncmers is most marked at high compression and high error rates, as is expected due to their higher conservation. Yet the advantage is already manifest at the lower compression rates commonly used by existing mappers. For large genomes, such as the human genome, using the higher compression enabled by syncmers also leads to lower RAM usage. Using the PSS with the best *ℓ*_2,*mut*_ also improves over closed syncmers due to their better distribution. Syncmer-minimap is slower than the highly optimized minimap2, taking 50–100% longer to map reads, but it is faster than Winnowmap2. Future work should focus on lowering the runtime by optimizing the syncmer mapping implementation.

The well-developed minimap2 and Winnowmap2 software tools have a variety of internal parameters, and by adjusting them one may be able to achieve some of the performance advantage of PSS. The approach we propose here is more principled and avoids the need to guess or grid search across parameters in order to get the best mapping performance. Increased performance achieved in such a manner could likely improve the syncmer mappers as well, as improving the choice of alignment seeds is orthogonal to many of the other algorithmic details of read mapping.

There are a number of issues and questions that this work leaves open, particularly in the theoretical analysis. First, the analysis of windowed schemes and downsampled schemes under mutation remains to be completed. Second, an expression for *ℓ*_2_ for minimizer schemes could also be obtained. Third, can the theory be expanded to canonical *k*-mers? Fourth, it would be helpful to obtain more robust definitions of conservation and *ℓ*_2_ that do not depend on preserving indices between sequences, thereby allowing indels to be included in the theoretical analysis. Finally, what is the ideal metric for evaluating the performance of schemes? While we argue that *ℓ*_2_ is preferable to *ℓ*, other new metrics may capture mapping performance even more accurately.

Another possible avenue to explore is in the definition of the selection scheme itself. Is it possible to select *k*-mers in a biased way in order to increase the compression but still retain the beneficial distance distribution of syncmer schemes? Or could a sequence-specific set of *k*-mers be determined efficiently for any desired compression rate? The quest for a “best” selection scheme is not over.

## Supporting information

S1 Text Supplementary information, figures and tables. (PDF)

S1 Data Supplementary data tables A-I. (XLSX)
