# Supplementary material for: Parameterized syncmer schemes improve long-read mapping
Source: PLoS Comput Biol. 2022 Oct 28;18(10):e1010638. doi: 10.1371/journal.pcbi.1010638 (PMC9645665; doi:10.1371/journal.pcbi.1010638)
Supplement: S1 Text — (PDF) [file pcbi.1010638.s001.pdf]

Supplementary information for
Parameterized syncmer schemes improve long-read mapping

Abhinav Dutta*, David Pellow*, and Ron Shamir

October 19, 2022

A Definitions of downsampled and windowed syncmer schemes

Downsampled syncmers: Given a uniformly random hash function $h : \Sigma^k \rightarrow [0, H]$, for a given string S, *downsampling* selects syncmers only from the set of $|\Sigma|^k/\delta$ k-mers with the lowest hash values.

$$\mathcal{DS}_{k,s,o,\{x_1,...,x_n\},h,\delta}(S) = \{i | i \in \mathbb{S}_{k,s,o}(x_1, ..., x_n)(S) \wedge h(S[i, i + k - 1]) < H/\delta\}$$

We call δ the *downsampling rate*.

Windowed syncmers: Windowed syncmers fill in gaps using a minimizer scheme, thus providing a window guarantee. For clarity in the definition below let $\mathbb{S}(S)$ represent $\mathbb{S}_{k,s,o}(x_1, ..., x_n)(S)$.

$$f_{k,s,w,o,\{x_1,...,x_n\}}(S) = \left\{i | i \in \mathbb{S}(S) \bigcup i \in \mathcal{M}_{k,w,o}(S[j, j + w - 1]) \; \forall j \; s.t. \; \mathbb{S}(S[j, j + w - 1]) = \emptyset\right\}$$

Letting $\mathcal{S}$ represent all k-mers that can be syncmers in $\mathbb{S}_{k,s,o}(x_1, ..., x_n)$, an equivalent definition would be: $\mathcal{M}_{k,w,o'}(S)$ where o' is defined such that $x \in \mathcal{S}, x' \in \Sigma^k \setminus \mathcal{S} \implies x < x'$.

B Analysis of parameterized syncmer schemes

In this section we provide our full theoretical analysis of PSS.

B.1 Recursive expressions for conservation of PSS

Consider a window of α consecutive k-mers. We assume random sequence (i.e., made up of iid bases) throughout. Let s_β be the s-minimizer in the α-window, at position β. Then if t is a parameter of the syncmer scheme, s_β generates a syncmer if it is not in the first $t - 1$ or last $k - t$ positions in the α-window. If β is not in a position where it generates a syncmer, we recursively check to the left or right of β to see if a syncmer is generated by the s-minimizer of that region. See Fig **??** for an example.

For a 1-parameter scheme f with k-mer length k, s-minimizer length s, and parameter t let $P(\alpha)$ be the probability of selecting at least one syncmer in a window of α adjacent k-mers. Then, assuming a uniformly random hash over the s-mers, and conditioning on the position of the s-minimizer of the α-window, β:

$$P(\alpha) = p_\beta \sum_\beta P(\alpha|\beta) \approx \frac{1}{k + \alpha - s} \left[\sum_{\beta=1}^{t-1} P(\alpha - \beta) + \sum_{\beta=t}^{t+\alpha-1} 1 + \sum_{t+\alpha}^{k+\alpha-s} P(\beta - k + s - 1) \right].$$

The probability of any of the $k + \alpha - s$ starting positions being the s-minimizer is denoted as p_β and assumed to be uniform. This assumption starts to break down when the s-minimizer is not unique, thus we note that the probability is approximate. If β is in the first $t - 1$ or last $k - t$ starting positions (red regions in Fig AA), then a syncmer may be generated by the remaining $\alpha - \beta$ positions to the right or $\beta - k + s - 1$ positions to the left, respectively. Note we define $\sum_{x=i}^{j} = 0$ when $i > j$ and $P(x) = 0$ when $x \leq 0$.

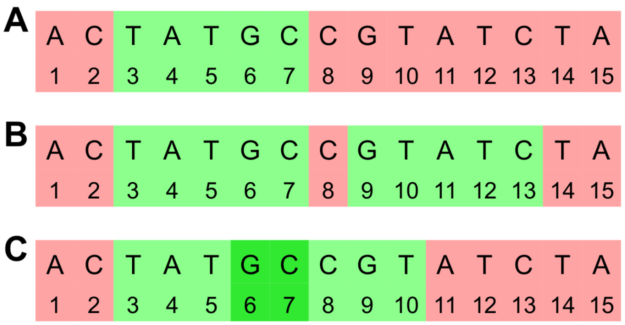

Fig A Illustration of s-minimizers generating syncmers. A window of $\alpha = 5$ consecutive 11-mers. **A**: When $s = 5$ and $t = 3$, then the s-minimizer of the entire window generates a syncmer when its starting index is in the green region. If the s-minimizer is in one of the red regions then a syncmer may be generated by the s-minimizer of the remaining part of the window. For a two parameter scheme the s-minimizer creates two syncmer generating regions that may be disjoint (**B**) if $s > t_2 - t_1$ or overlapping (**C**) if $s < t_2 - t_1$. In this example, $t_1 = 3$ and $t_2 = 9$ in **B** and $t_2 = 6$ in **C**.

When downsampling syncmers, there is a probability of $1/\delta$ that an s-minimizer in the syncmer generating region (i.e. the green region in Fig AA) really generates a syncmer. If it does not, then the left and right regions are considered recursively, yielding the following expression, where we simplify notation by letting $P(\alpha - \beta) = P_R$ and $P(\beta - k + s - 1) = P_L$:

$$P(\alpha) \approx \frac{1}{k+\alpha-s}\left[\sum_{\beta=1}^{t-1} P_R + \sum_{\beta=t}^{t+\alpha-1}\left(\frac{1}{\delta} + (1-\frac{1}{\delta})\,(P_R + P_L - P_R \cdot P_L)\right) + \sum_{t+\alpha}^{k+\alpha-s} P_L\right].$$

In the case of 2-parameter schemes, two syncmers are generated by s_β in regions that will overlap if the parameters t_1 and t_2 are within s of each other, and will be disjoint otherwise (see Fig AB,C). Combining these two cases into a single recursive expression yields:

$$P(\alpha) \approx \frac{1}{k+\alpha-s} \cdot \left[\sum_{\beta=1}^{t_1-1} P_R + \sum_{\beta=t_1}^{\substack{min \\ (t_2-1,t_1+\alpha-1)}} 1 + \sum_{\substack{\beta=min \\ (t_2,t_1+\alpha)}}^{t_1+\alpha-1} 1 + \sum_{\substack{\beta=min \\ (t_2,t_1+\alpha)}}^{t_2-1} (P_R + P_L) + \sum_{\substack{\beta=max \\ (t_1+\alpha,t_2)}}^{t_2+\alpha-1} 1 + \sum_{t+\alpha}^{k+\alpha-s} P_L\right].$$

When downsampling is used then the 1 in the second and fifth sums is replaced by $\frac{1}{\delta}+(1-\frac{1}{\delta})\,(P_R + P_L - P_R \cdot P_L)$ as in the one parameter case. The third sum expresses the overlapped region where either parameter creates a syncmer, when it exists. When *both* generated syncmers are downsampled then the left and right sides are recursively checked, thus the 1 is replaced by $(1-(1-\frac{1}{\delta})^2 + (1-\frac{1}{\delta})^2\,(P_R + P_L - P_R \cdot P_L)$.

This expression can be greatly simplified by introducing the notation $count(\beta)$ that represents the number of syncmers generated by the s-minimizer s_β. For example, $count(\beta) = 0$ in the red region of Fig A and $count(\beta) = 2$ in the overlapped region when $\beta = 6$ or 7 in Fig AC. The general expression for $P(\alpha)$ for *any* PSS is:

$$P(\alpha) \approx \frac{1}{k+\alpha-s} \cdot \left[\sum_{\beta=1}^{k+\alpha-s}\left(1-(1-\frac{1}{\delta})^{count(\beta)}\right) + (1-\frac{1}{\delta})^{count(\beta)}\,(P_R + P_L - P_R \cdot P_L)\right].$$

Note that this definition relies on the definition $P(x) = 0, x \leq 0$ to include the correct terms for the correct values of β.

The value of $P(\alpha)$ can thus be computed efficiently for any PSS and used to calculate the conservation using the formula from [1].

B.2 Calculating the distance distribution

For a given scheme, the distribution of distances between adjacent syncmer positions is specified by $Pr(d = x)$, the probability that the distance d is x. To calculate this probability, we define the new quantities $F(\alpha)$ and $L(\alpha)$ denoting the probability that *only* the first or *only* the last k-mer in a window of α k-mers is a syncmer, respectively. We refer to these k-mers as K_1 and K_α respectively. Note that due to symmetry $F(\alpha) = L(\alpha)$. Note also that $1 - P(\alpha)$ gives the probability that *no* k-mer in an α-window is a syncmer.

We compute $F(\alpha)$ by conditioning on β as before. For simplicity we divide the sum over β into cases based on the syncmers that are generated by s_β rather than breaking up the sum across different values of β. With some abuse of notation, we let K_i represent the event the that s_β generates K_i as a syncmer.

$$F(\alpha) \approx \frac{1}{k+\alpha-s} \sum_{\beta=1}^{k+\alpha-s} \begin{cases} \frac{1}{\delta} \cdot (1-\frac{1}{\delta})^{count(\beta)-1} \cdot (1 - P(\alpha-\beta)) & K_1 \\ (1-\frac{1}{\delta})^{count(\beta)} \cdot F(\beta-k+s-1) \cdot (1-P(\alpha-\beta)) & \text{otherwise} \end{cases}$$

In the first case we have the probability that K_1 is not downsampled, any other syncmer generated by s_β is downsampled, and there are no other syncmers generated to the right of β. In the second case we have the probability that any syncmers generated by s_β are downsampled, no syncmers are generated to the right of β, and the recursive computation of the probability that the s-minimizer of the segment to the left of β generates a syncmer at K_1.

Similarly, define $D(\alpha)$ to be the probability that in a window of α k-mers *only* the first *and* last k-mers are syncmers. Then

$$D(\alpha) \approx \frac{1}{k+\alpha-s} \sum_{\beta=1}^{k+\alpha-s} \begin{cases} (\frac{1}{\delta})^2 \cdot (1-\frac{1}{\delta})^{count(\beta)-2} & K_1, K_\alpha \\ \frac{1}{\delta} \cdot (1-\frac{1}{\delta})^{count(\beta)-1} \cdot F(\alpha-\beta) & K_1, \neg K_\alpha \\ \frac{1}{\delta} \cdot (1-\frac{1}{\delta})^{count(\beta)-1} \cdot F(\beta-k+s-1) & K_\alpha, \neg K_1 \\ (1-\frac{1}{\delta})^{count(\beta)} \cdot F(\beta-k+s-1) \cdot F(\alpha-\beta) & \text{otherwise} \end{cases}$$

B.3 Calculating $\ell_{2,mut}$

To compute the desired metric $\ell_{2,mut}$ we must calculate the metrics from the previous section but only with *conserved* syncmers. We add the subscript '*mut*' to a value to indicate that only conserved syncmers are considered. The impact of mutations is similar to that of downsampling shown in the previous section, except that when a syncmer is lost due to mutation, the surrounding k-mers are also lost. In this case we consider no downsampling to make the expressions simpler.

Let Ω_β be the set of syncmers generated by s_β, and ω_{β_i} be the members of this set. Note that $|\Omega_\beta| = count(\beta)$. Then,

$$P_{mut}(\alpha) \approx \frac{1}{k+\alpha-s} \sum_{\beta=1}^{k+\alpha-s} \left(Pr(\exists \text{ conserved } \omega_{\beta_i} \in \Omega_\beta) + Pr(\nexists \text{ conserved } \omega_{\beta_i} \in \Omega_\beta, \exists \text{ syncmer to the left or right})\right)$$

For convenience we call the first probability $P_{conserved}$ and the second $P_{recurse}$.

$P_{conserved}$ is computed using the inclusion-exclusion principle:

$$P_{conserved} = \sum_i Pr(\omega_i \text{ conserved}) - \sum_{i<j} Pr(\omega_i, \omega_j \text{ conserved}) + \sum_{i<j<k} Pr(\omega_i, \omega_j, \omega_k \text{ conserved}) - ...$$

Every term in this series is calculated as $(1-\theta)^{countBases}$ where θ is the mutation rate and *countBases* counts the number of bases covered by the conserved k-mers (i.e. if two conserved syncmers overlap, the shared bases are counted only once).

$P_{recurse}$ is more complicated. We again sum over all values of β. When Ω_β is empty (e.g. β is in the red region), then the recursion is similar to the case without mutation. Otherwise, all of the syncmers are lost

due to mutation, and we additionally sum over the possible positions of the first and last points of mutation in Ω_β, named f and l, respectively.

$$P_{recurse} \approx \frac{1}{k+\alpha-s} \times \sum_{\beta=1}^{k+\alpha-s} \begin{cases} P_{mut}(\alpha-\beta) + P_{mut}(\beta-k+s-1) - P_{mut}(\alpha-\beta) \cdot P_{mut}(\beta-k+s-1) & \Omega_\beta = \emptyset \\ \sum_{f \le l} Pr(\nexists \text{ conserved } \omega_\beta \in \Omega_\beta, f, l) \times (P_{mut}(left) + P_{mut}(right) - P_{mut}(left) \cdot P_{mut}(right)) & \text{otherwise} \end{cases}$$

Here $left = max(0, min(f-k, \beta-k+s-1))$ and $right = max(0, min(\alpha-l, \alpha-\beta))$. We expand the joint probability as

$$Pr(\nexists \text{ conserved } \omega_\beta \in \Omega_\beta, f, l) = \theta^y \cdot (1-\theta)^x \cdot Pr(\nexists \text{ conserved } \omega_\beta \in \Omega_\beta | f, l)$$

where y is 1 if $l = f$ and 2 otherwise, and x is the number of unmutated bases that is fixed by the given values of f and l. The conditional probability is written as

$$Pr(\nexists \text{ conserved } \omega_\beta \in \Omega_\beta | f, l) = 1 - Pr(\exists \text{ conserved } \omega_\beta \in \Omega_\beta | f, l)$$

and is computed using the inclusion-exclusion principle as above.

$F(\alpha)$ and $D(\alpha)$ are extended to the case of mutation similarly:

$$F_{mut}(\alpha) \approx \frac{1}{k+\alpha-s} \sum_{\beta=1}^{k+\alpha-s} \begin{cases} \sum\limits_{f \le l} Pr(\nexists \text{ conserved } \omega_\beta \in \Omega_\beta \setminus K_1, K_1 \text{ conserved}, f, l) \cdot (1 - P_{mut}(right|b)) & K_1, \Omega_\beta \setminus K_1 \neq \emptyset \\ Pr(K_1 \text{ conserved}) \cdot (1 - P_{mut}(\alpha-\beta|b)) & K_1, \Omega_\beta \setminus K_1 = \emptyset \\ \sum\limits_{f \le l} Pr(\nexists \text{ conserved } \omega_\beta \in \Omega_\beta, f, l) \cdot (1 - P_{mut}(right|b)) \cdot F_{mut}(left|b') & \neg K_1, \Omega_\beta \neq \emptyset \\ F_{mut}(\beta-k+s-1) \cdot (1 - P_{mut}(\alpha-\beta)) & \text{otherwise} \end{cases}$$

$$D_{mut}(\alpha) \approx \frac{1}{k+\alpha-s} \times \sum_{\beta=1}^{k+\alpha-s} \begin{cases} \sum\limits_{f \le l} Pr(\nexists \text{ conserved } \omega_\beta \in \Omega_\beta \setminus K_1, K_1 \text{ conserved}, f, l) \cdot F_{mut}(right|b) & K_1, \neg K_\alpha, \Omega_\beta \setminus K_1 \neq \emptyset \\ (1-\theta)^k \cdot F_{mut}(\alpha-\beta|b) & K_1, \neg K_\alpha, \Omega_\beta \setminus K_1 = \emptyset \\ \sum\limits_{f \le l} Pr(\nexists \text{ conserved } \omega_\beta \in \Omega_\beta \setminus K_\alpha, K_\alpha \text{ conserved}, f, l) \cdot F_{mut}(left|b) & K_\alpha, \neg K_1, \Omega_\beta \setminus K_\alpha \neq \emptyset \\ (1-\theta)^k \cdot F_{mut}(\beta-k+s-1|b) & K_\alpha, \neg K_1, \Omega_\beta \setminus K_\alpha = \emptyset \\ \sum\limits_{f \le l} Pr(\nexists \text{ conserved } \omega_\beta \in \Omega_\beta \setminus \{K_1 \cup K_\alpha\}, \{K_1 \cup K_\alpha\} \text{ conserved}, f, l) & K_1, K_\alpha, \Omega_\beta \setminus \{K_1 \cup K_\alpha\} \neq \emptyset \\ Pr(\{K_1 \cup K_\alpha\} \text{ conserved}) & K_1, K_\alpha, \Omega_\beta \setminus \{K_1 \cup K_\alpha\} = \emptyset \\ \sum\limits_{f \le l} Pr(\nexists \text{ conserved } \omega_\beta \in \Omega_\beta, f, l) \cdot F_{mut}(right) \cdot F_{mut}(left|b) & \neg K_1, \neg K_\alpha, \Omega_\beta \neq \emptyset \\ F_{mut}(\alpha-\beta) \cdot F_{mut}(\beta-k+s-1) & \neg K_1, \neg K_\alpha, \Omega_\beta = \emptyset \end{cases}$$

Here we again divide into cases depending on whether there are syncmers that can be lost. We have also used recursive expressions that are similar to the above except we are given that b bases to the left or right of the defined region are conserved. These are calculated using similar techniques as above.

Finally, we can use these expressions to compute:

$$\ell_{mut} = \sum_{x=k+1}^{\infty} (x-k) \cdot D_{mut}(x+1)$$

$$\ell_{2,mut} = \sqrt{\sum_{x=k+1}^{\infty} (x-k)^2 \cdot D_{mut}(x+1)}$$

Scheme	Theoretical	Simulated
$\mathbb{S}(3,4)$	14.2717	14.3307
$\mathbb{S}(3,5)$	13.2935	13.317
$\mathbb{S}(3,6)$	12.6868	12.6947
$\mathbb{S}(3,7)$	12.3342	12.3426
$\mathbb{S}(3,8)$	12.1713	12.1776
$\mathbb{S}(3,9)$	12.1631	12.1809
$\mathbb{S}(3,10)$	12.2915	12.3076
$\mathbb{S}(3,11)$	12.5477	12.5702

Table A Comparison of theoretical and simulated values of $\ell_{2,mut}$. Values of $\ell_{2,mut}$ for a selection of schemes with $k = 15$, $s = 5$, $\theta = 0.15$. Theoretical values were computed using 1000 terms. Simulated values were found on a simulated sequence of length 50,000,000.

Note that, unlike $P(\alpha)$, which can be computed efficiently, the computation of these metrics includes an infinite sum. The sum can be truncated at an appropriate distance x, however there are still many more terms than in the computation of the conservation. In practice, simulating a very long sequence, selecting syncmers, and simulating mutations to determine these metrics empirically is much less time consuming and yields results that are very close to the true values. We used this simulation method to compute $\ell_{2,mut}$ for $k = 11, 13, 15, 17$ and 19, mutation rates $0.05, 0.15$ and 0.25, and all 2- and 3-parameter schemes Results are presented in Supplementary Data File 1, Table SD2 (note that for 1-parameter schemes the best ℓ_2 and ℓ are the same, and thus already known from [1]). The computed theoretical expressions for some parameter combinations are available in Supplementary Data File 1, Table SD3. A comparison of the values computed theoretically and by simulation for the 2-parameter schemes $\mathbb{S}(3, x)$, $x \in [4, 11]$ with $k = 15$, $s = 5$, 15% mutation rate can be seen in Table A.

C Windowed PSS implementation

Algorithm A describes the implementation of windowed PSS.

Algorithm A Windowed syncmer selection

Input: Sequence S, syncmer parameters x_1,x_2, ...x_n, k-mer length k, s-mer length s, window w, downsampling rate δ (default: 1)

Output: P, a list of selected positions.

$P \leftarrow \{\}$
for $j \in 1$ to $|S| - w + 1$ **do**
  $hasSync \leftarrow$ FALSE
  for $l \in 0$ to $w - k$ **do**
    $kmer = Canonical_{h_1}(S[j + l, j + l + k - 1])$
    $m = \operatorname{argmin}_{t \in [0,k-s]} h_2(kmer[t, t + s])$
    if $m \in \{x_1, x_2..., x_n\}$ **and** $h_2(kmer) < 1/\delta$ **then**
      $P \leftarrow P \cup \{j + l\}$
      $hasSync \leftarrow$ TRUE
  if $hasSync$ **is** FALSE **then**
    $m = \operatorname{argmin}_{l \in [0,w-k]} h_2(Canonical_{h_1}(S[j + l, j + l + k - 1]))$
    $P \leftarrow P \cup \{j + m\}$
return P

D Syncmer based mapping implementations

Modifications to the mappers were minimal. Only the code that selects the k-mers to use as seeds to index the reference sequence and in the query reads was modified. Here we describe implementation details and optimizations in the code that differ from the high-level descriptions in Algorithm **??** and A.

In minimap2 the most frequent minimizers (0.02% by default) are dropped to reduce spurious matches and lower the runtime and memory usage. We also drop the most frequent selected k-mers as the last stage of all minimap syncmer variants for consistency. In Winnowmap, the most frequent k-mers (also 0.02% by default) are re-weighted in the minimizer order so they are less likely to be selected as minimizers. In the syncmer-winnowmap variant, we do not consider k-mer weighting, and thus we simply drop these k-mers if they are selected. However, in the *windowed* syncmer-winnowmap variant we do re-weight the frequent k-mers before selecting minimizers in empty windows.

We use several different hashes in our syncmer variants of the mappers: h_{can} to select canonical k-mers, h_s to select s-minimizers, h_{min} to select minimizers for windowed variants and h_{down} for downsampling. We require that $h_{can} \neq h_{down}$ to maintain random downsampling. In minimap syncmer variants we use hash64 from minimap2 for h_{min} and a variant of MurmurHash2 that ensures $murmur2(0) \neq 0$ to ensure randomness for the other hashes. Thus $h_{min} = -hash64/\text{UINT64\_MAX}$, $h_s(x) = murmur2(x)$, $h_{down}(x) = murmur2(x)$, and $h_{can}(x) = murmur2(x << 1 + 5)$ to ensure that it has a different value than h_{down}. For winnowmap variants we use $h_{can}(x) = lexicographic(x)$ as this is what is used by the k-mer counter Meryl, $h_{min}(x) = -(murmur2(x)/\text{UINT64\_MAX})^8$ in the case that the minimizer is one of the most frequent and $-murmur2(x)/\text{UINT64\_MAX}$ otherwise. The other hashes are as in the minimap variants.

In all windowed variants, downsampling occurs before filling in empty windows with minimizers.

ONT reads were mapped using the `map-ont` option in all mappers, while PacBio reads were mapped using the `map-pb` option (`map-pb-clr` in Winnowmap and variants). The latter uses homopolymer compression (HPC) and thus has a real compression (on the non-HPC sequence) that is above the theoretical one. In Winnowmap2 the `map-ont` option uses SV mode that attempts to resolve structural variation and may affect performance.

E Mapper command line arguments

The command line arguments of syncmer-minimap and syncmer-winnowmap extend the definitions of the original tools. minimap2 and Winnowmap2 were called as follows:

```
./minimap2 -x [map-pb|map-ont] -w <w> -k <k> -t <# threads> -a <ref>  <reads>

./winnowmap -W <repeats file> -x [map-pb-clr|map-ont] [-H] -w <w> -k <k> -t <# threads>
           -a <ref>  <reads>
```

The options `map-pb`, `map-pb-clr` and `-H` are used for PacBio reads and `map-ont` for ONT reads. For the syncmer versions, command line options are added for the PSS parameters (positions of the s-minimizer), s-minimizer length, and the downsampling rate:

```
./minimap2 -x [map-pb|map-ont] --downsample <d> --s-mer <s> --pos1 <x1>
          [--pos2 <x2> [--pos3 <x3> [--pos4 <x4>]]] [-w <w>] -k <k> -t <# threads> -a <ref>  <reads>

./winnowmap -W <repeats file> -x [map-pb-clr|map-ont] [-H] --downsample <d> --s-mer <s>
           --pos1 <x1> [--pos2 <x2> [--pos3 <x3> [--pos4 <x4>]]] [-w <w>] -k <k> -t <# threads>
           -a <ref>  <reads>
```

Note that in the syncmer mappers the option `-w` is used to define windowed syncmer schemes and is set to 0 for no windowing by default.

F Simulation details

PacBio reads were simulated using PBSim [2] with the CLR model and the following parameter settings: depth 10, mean length 9000, length std 6000, minimum length 100, maximum length 40000, mean accuracy 0.87, accuracy std 0.02, minimum accuracy 0.85, maximum accuracy 1, and difference ratio 10:48:19. Error rates and read lengths roughly matched to the statistics observed in a recent benchmark of long read correction methods [3] unless otherwise indicated.

ONT reads were simulated using NanoSim [4] with default parameters and the human pre-trained model for Guppy base calls.

To evaluate mapping correctness for the PacBio simulated data we used the mapeval utility of paftools packaged with minimap2. In this tool, reads are considered correctly mapped if the overlap between the read alignment and the true read location is $\geq 10\%$ of the combined length of the true read and aligned read interval. This criterion was also used in [5].

G Bacterial species used

We chose a single representative assembly of each strain from [6] with the fewest, longest and most highly covered contigs and concatenated all references into a single fasta file. Reads from the same samples were downloaded. Assemblies and reads from following samples were used:

- bc1019, Bacillus cereus 971 (ATCC 14579)
- bc1059, Bacillus subtilis W23
- bc1101, Burkholderia cepacia (ATCC 25416)
- bc1102, Enterococcus faecalis OG1RF (ATCC 47077D-5)
- bc1111, Escherichia coli K12
- bc1087, Escherichia coli W (ATCC 9637)
- bc1018, Helicobacter pylori J99 (ATCC 700824)
- bc1077, Klebsiella pneumoniae (ATCC BAA-2146)
- bc1082, Listeria monocytogenes (ATCC 19117)
- bc1043, Methanocorpusculum labreanum Z (ATCC 43576)
- bc1047, Neisseria meningitidis FAM18 (ATCC 700532)
- bc1054, Rhodopseudomonas palustris
- bc1119, Staphylococcus aureus HPV (ATCC BAA-44)
- bc1079, Staphylococcus aureus subsp. aureus (ATCC 25923)
- bc1052, Treponema denticola A (ATCC 35405)

H Performance of minimizers and PSS on real genome sequences without mutations and with low mutation rate

The metrics measured on real genomes without mutations are shown in Table B.

The metrics are shown for the low mutation rate of 4% in Table C. The optimal PSS outperforms minimizers even at this low mutation rate, but not at lower rates.

I Results when using forward-only (non-canonical) k-mers

We tested the performance metrics on real genomes when using only forward k-mers and not canonical ones. Table D shows the metrics when only forward k-mers are used. The ranking between schemes is unchanged and the values of the metrics remain similar.

To test the impact of using canonical k-mers on the distance distribution between selected positions we used $\mathbb{S}_{15,5}(3, 9)$ on the unmutated CHM13X. Fig BA shows the distance distribution for syncmers selected only

Dataset	Scheme	Compression	ℓ	ℓ_2	$p90$	$p100$	# positions
ECK12	$\mathcal{M}_{15,10}$	5.503	3.23×10^{-6}	0.005	9	10	843,408
	$\mathbb{S}_{15,5}(3,9)$	5.490	0.0191	0.377	10	53	845,419
	$\mathbb{S}_{15,5}(1,11)$	5.509	0.0255	0.443	11	52	842,557
	$\mathbb{S}_{15,5}(1,2)$	5.502	0.0566	0.715	13	66	843,634
	$\mathcal{M}_{15,19}$	9.989	0.0527	0.398	18	19	464,660
	$\mathbb{S}_{15,6}(6)$	9.986	0.133	1.375	19	99	464,810
CHM13X	$\mathcal{M}_{15,10}$	5.489	5.83×10^{-8}	0.0005	9	10	28,099,399
	$\mathbb{S}_{15,5}(3,9)$	5.523	0.0205	0.412	10	161	27,930,897
	$\mathbb{S}_{15,5}(1,11)$	5.437	0.0259	0.449	11	260	28,371,548
	$\mathbb{S}_{15,5}(1,2)$	5.350	0.0549	0.706	13	122	28,835,008
	$\mathcal{M}_{15,19}$	9.977	0.0526	0.3974	18	19	15,461,458
	$\mathbb{S}_{15,6}(6)$	10.055	0.137	1.419	20	735	15,342,238

Table B Properties of minimizer and syncmer schemes on real sequences without mutations. The best syncmer schemes with theoretical compression of 5.5 and 10 were chosen. The table shows the actual compression and other metrics on the real sequences, # positions is the number of positions that were selected by the scheme.

Dataset	Scheme	Compression	ℓ	ℓ_2	$p90$	$p100$	# conserved
ECK12	$\mathcal{M}_{15,10}$	10.443	0.312	3.048	26	225	1,353,736
	$\mathbb{S}_{15,5}(3,9)$	9.795	0.308	3.012	25	205	1,443,355
	$\mathbb{S}_{15,5}(1,11)$	9.708	0.327	3.181	26	235	1,456,277
	$\mathbb{S}_{15,5}(1,2)$	9.601	0.365	3.517	27	214	1,472,511
CHM13X	$\mathcal{M}_{15,10}$	10.030	0.294	2.896	25	240	15,379,308
	$\mathbb{S}_{15,5}(3,9)$	9.499	0.292	2.874	24	220	16,238,925
	$\mathbb{S}_{15,5}(1,11)$	9.344	0.310	3.030	25	260	16,508,520
	$\mathbb{S}_{15,5}(1,2)$	9.187	0.348	3.365	26	266	16,790,565

Table C Properties of minimizer and syncmer schemes on real sequences with low mutation rate. Substitutions were introduced in the references at a rate of 4%. The values shown are for the conserved selected k-mers. # conserved is the number of k-mers selected by a scheme that were conserved under mutation.

Dataset	Scheme	Description	Compression	ℓ	ℓ_2	$p90$	$p100$	# conserved
ECK12	$\mathcal{M}_{15,10}$	minimap minimizer	75.86	0.86	13.61	208	1323	61,184
	$\mathbb{S}_{15,5}(3,9)$	optimal PSS	62.14	**0.83**	**12.10**	**171**	**947**	74,695
	$\mathbb{S}_{15,5}(1,11)$	closed syncmer	62.59	0.85	12.99	181	1046	74,158
	$\mathbb{S}_{15,5}(1,2)$	“bad PSS”	62.09	0.88	15.04	200	1278	74,756
CHM13X	$\mathcal{M}_{15,10}$	minimap minimizer	54.27	0.81	11.70	152	1424	2,842,246
	$\mathbb{S}_{15,5}(3,9)$	optimal PSS	45.41	**0.77**	**10.41**	**126**	**1219**	3,397,027
	$\mathbb{S}_{15,5}(1,11)$	closed syncmer	44.83	0.80	11.15	131	1173	3,440,892
	$\mathbb{S}_{15,5}(1,2)$	“bad PSS”	43.64	0.84	12.89	142	1481	3,534,565

Table D Performance metrics of minimizer and syncmer schemes using *non-canonical* k-mers on real sequences with simulated mutations. Substitutions were introduced in the references at a rate of 15%. The values shown are for the conserved selected k-mers. # conserved is the number of k-mers selected by a scheme that were conserved under mutation. Best performance is shown in bold.

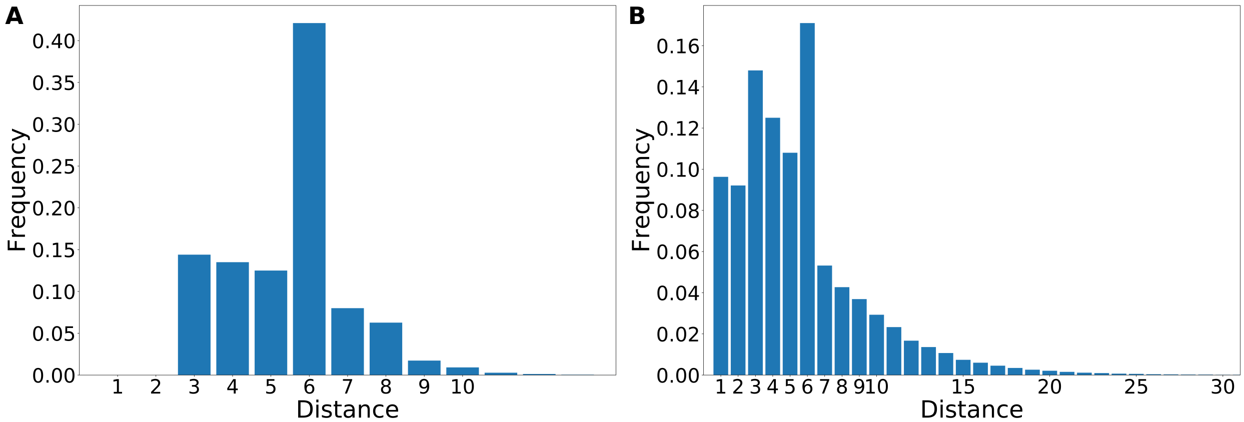

Fig B Distribution of the distances between selected positions in a syncmer scheme. The distribution of distances between consecutive selected positions of syncmer scheme $\mathbb{S}_{15,5}(3,9)$ on the CHM13X reference is shown. **A.** The distribution of syncmers selected only in the forward orientation. **B.** Canonical syncmers. For visualization purposes the distribution is shown only for distances with frequency $> 10^{-5}$. The true maximum distance is 161 for canonical k-mers (see Supplementary Table B) and 76 for the forward k-mers, but the frequency of the longer distances is extremely low.

using forward strand k-mers. The minimum distance is 3 and there is a sharp peak at 6. In general, for a scheme with parameters $x_1 < ... < x_n$, the minimum distance is $min(x_1, k - s - x_n + 2)$ and there are peaks at $x_{i+1} - x_i$, in agreement with the expression for $D(\alpha)$ above (**??**). In mapping, read orientations are unknown and canonical syncmers are used. Fig BB shows the results using canonical k-mers. The distance distribution still retains the peak at 6 and a local maximum at 3, but now adjacent positions are selected, and it has a much longer tail of distances (compare Supplementary Table B). We conclude that while the theory is limited to single-stranded sequences it shows trends that hold for canonical k-mers.

To demonstrate that the improved mapping performance does not depend on the choice to use canonical k-mers, we disabled selection of seeds from the reverse strand in both indexing and read mapping. Results are shown in Figure C. Although many more reads are unmapped due to having selectable k-mers only in the reverse orientation, we see that the gap in mapping performance is similar to what was observed using canonical k-mers as seeds. In addition, the runtime of syncmer-minimap is much closer to that of minimap2, as expected since it no longer keeps track of the s-minimizers on two strands.

J Supplemental performance results

Fig D shows additional results for the number of unmapped reads at low, medium, and high compression rates.

Fig E compares the performance of the theoretically optimal 2-parameter PSS and closed syncmers in mapping real PacBio bacterial reads against the BAC reference.

K Windowed syncmer scheme results

Tables E and F present the properties of windowed syncmer schemes on real genome sequences with and without mutation, respectively.

Figures F and G present the number of unmapped reads and wrongly mapped reads for simulated datasets. These correspond to Figure **??** and include the results for windowed variants. Fig H presents the impact of percent sequence identity on the windowed variants as well, corresponding to Fig **??**.

Results on the real human and bacterial reads are presented in Fig I, and the runtimes and RAM usage for these runs are in Figures J and K. The runtime and memory usage on different tasks for the windowed

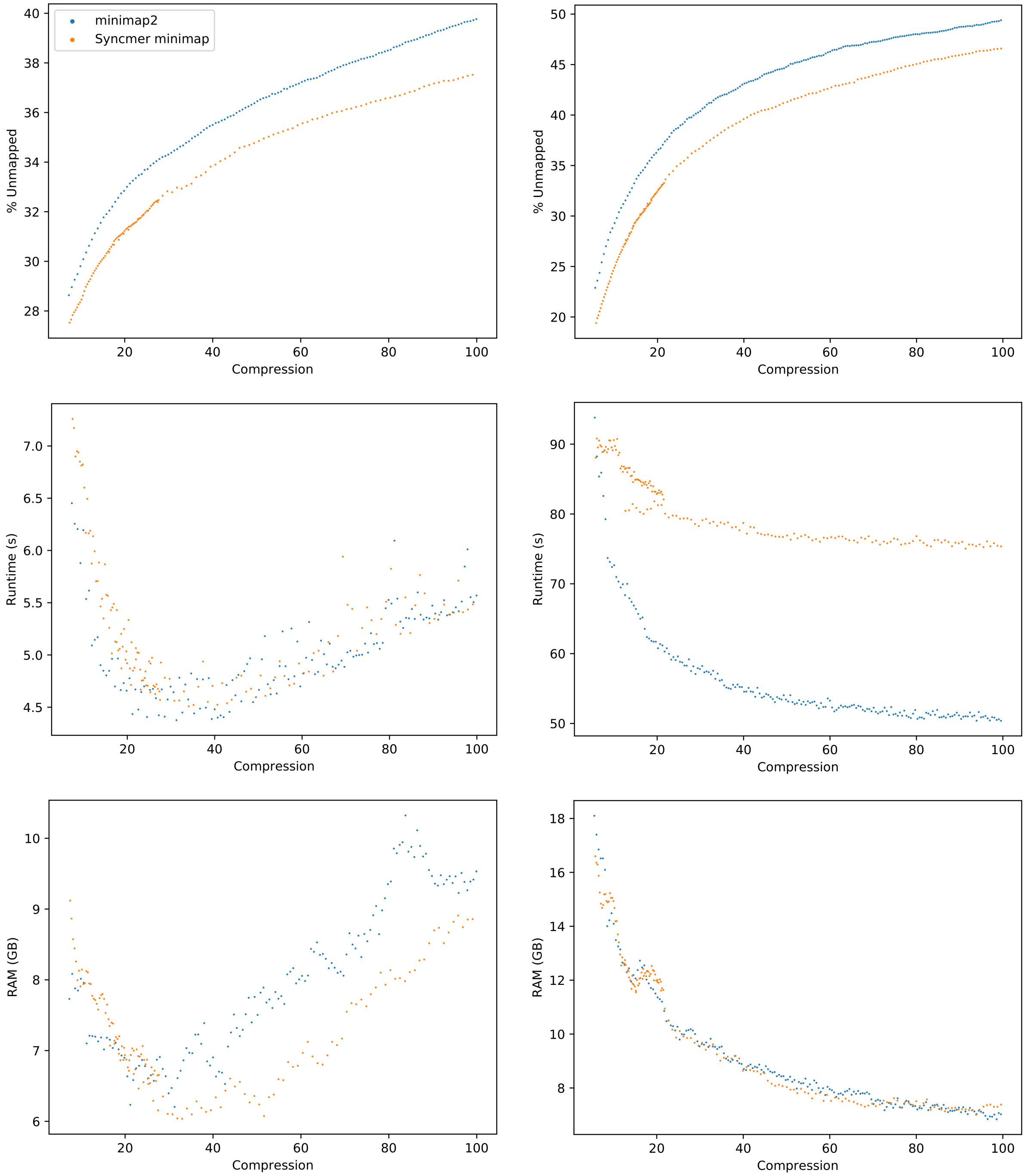

Fig C % unmapped, memory and runtime vs. compression using non-canonical k-mers.
Top: Percentage of unmapped reads. **Middle:** Runtime in seconds to index the reference and map reads by each method. **Bottom:** Peak RAM usage in GB to index the reference and map reads. **Left:** PacBio bacterial reads. **Right:** ONT human cell-line reads.

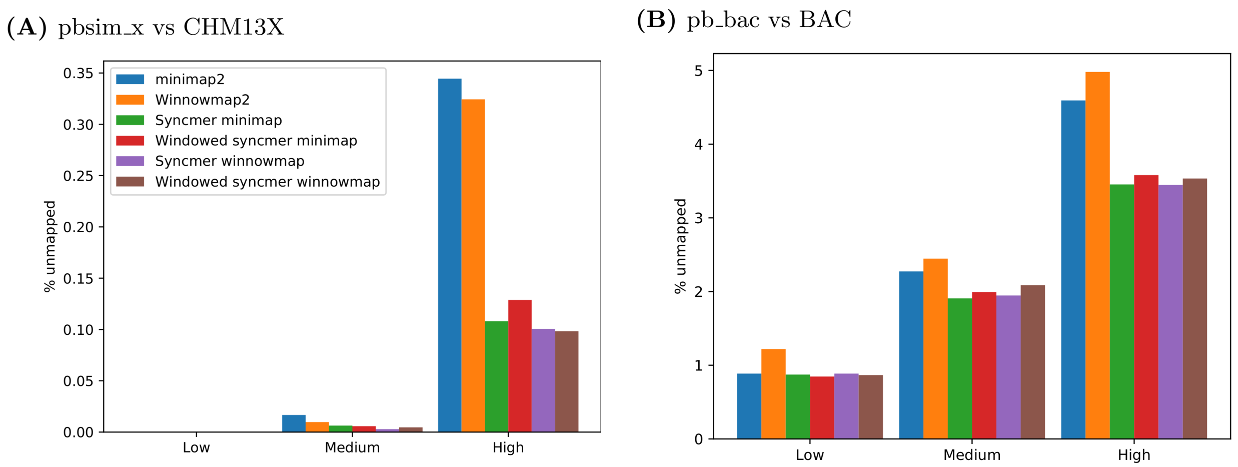

Fig D Percentage of unmapped reads – additional data. The percentage of unmapped reads is plotted for one simulated and one real read dataset mapped to their corresponding references. Minimizer and PSS parameters are as described in the Results section (The fraction of unmapped reads). **(A)** PacBio reads simulated from the CHM13 ChrX sequence mapped against CHM13X. Window sizes of windowed syncmer-minimap were $w = 13, 80, 165$ for the low, medium, and high compression variants, respectively, and for windowed syncmer-winnowmap they were $w = 14, 75, 170$, respectively. **(B)** 1000 PacBio reads sampled from each of the 15 bacterial species in BAC mapped against their reference genomes. Window sizes of windowed syncmer-minimap were $w = 13, 75, 175$ and in windowed syncmer-winnowmap they were $w = 16, 75, 170$ for the low, medium, and high compression variants, respectively.

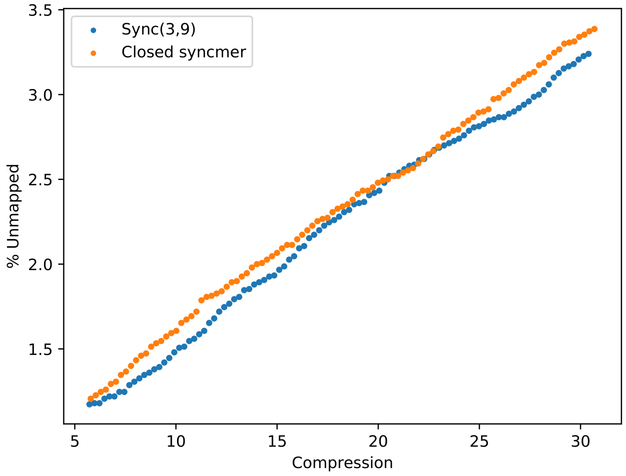

Fig E Comparison of 2-parameter PSS to closed syncmers. $\mathbb{S}(3, 9)$ and closed syncmers were used in syncmer-minimap to map PacBio bacterial reads against BAC. Both schemes used $k = 15$, $s = 5$.

w	δ	c	ℓ	ℓ_2	$p90$	$p100$	# positions	# syncmer	# minimizer
10	1	4.775	2.59×10^{-8}	0.000255	9	10	32,309,357	27,969,919	4,339,438
15	1	5.322	2.59×10^{-8}	0.000255	10	15	28,987,572	27,969,919	1,017,653
20	1	5.464	0.01047	0.1862	10	20	28,230,846	27,969,919	260,927
25	1	5.501	0.0167	0.3014	10	25	28,040,255	27,969,919	70,336
50	1	5.515	0.0197	0.3899	10	50	27,970,312	27,969,919	393
100	1	5.515	0.01973	0.3915	10	99	27,969,930	27,969,919	11
10	2	5.463	6.48×10^{-8}	0.000729	9	10	28,238,334	14,002,723	14,235,611
15	2	7.435	6.48×10^{-8}	0.000729	14	15	20,748,902	14,002,723	6,746,179
20	2	8.812	0.0493	0.4201	17	20	17,505,813	14,002,723	3,503,090
25	2	9.706	0.10495	0.8324	20	25	15,892,867	14,002,723	1,890,144
50	2	10.93	0.1986	1.788	23	50	14,112,958	14,002,723	110,235
100	2	11.015	0.2067	1.9811	23	100	14,003,934	14,002,723	1211

Table E Properties of windowed syncmers on CHM13X. Properties of windowed and down-sampled variants of $\mathbb{S}_{15,5}(3,9)$ are shown for a range of window lengths w on the human chromosome X sequence of CHM13. δ is the downsampling rate and c is the actual compression. The theoretical compression of the PSS (not downsampled) is 5.5. # positions is the number of positions that were selected by the scheme, # syncmers is the number of those that were selected by the PSS and # minimizers is the number of minimzers added to fill in gaps of length $\geq w$.

w	δ	c	ℓ	ℓ_2	$p90$	$p100$	# conserved	conserved syncmers	conserved minimizers
10	1	41.745	0.7826	10.713	122	1221	3,695,319	3,378,302	317,017
15	1	45.008	0.7928	11.041	131	1221	3,427,368	3,378,302	49066
20	1	45.558	0.7951	11.1125	132	1221	3,386,019	3,378,302	7717
25	1	45.646	0.7955	11.126	132	1221	3,379,466	3,378,302	1164
50	1	45.662	0.7956	11.129	133	1274	3,378,304	3,378,302	2
100	1	45.662	0.7956	11.128	133	1274	3,378,302	3,378,302	0
10	2	52.34	0.8062	11.515	147	1221	2,947,249	1,692,160	1,255,089
15	2	71.027	0.8397	12.898	190	1691	2,171,847	1,692,160	479,687
20	2	81.734	0.8572	13.774	216	1703	1,887,340	1,692,160	195,180
25	2	86.999	0.8655	14.257	230	1736	1,773,121	1,692,160	80961
50	2	91.104	0.8722	14.709	242	1736	1,693,222	1,692,160	1062
100	2	91.161	0.8723	14.718	242	1736	1,692,160	1,692,160	0

Table F Properties of conserved windowed syncmers under mutation. Properties of windowed and down-sampled variants of $\mathbb{S}_{15,5}(3,9)$ are shown for a range of window lengths w on CHM13X after simulating substitutions at a rate of 15%. w is the window size and δ is the downsampling rate. Properties of the conserved selected k-mers are reported.

(A) PacBio reads mapped to ChrX of GRCh

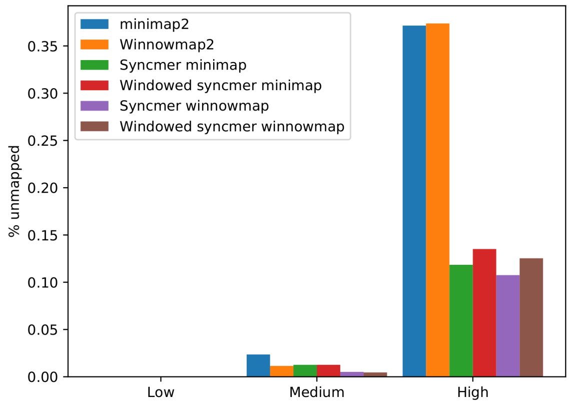

(B) ONT reads mapped to GRCh

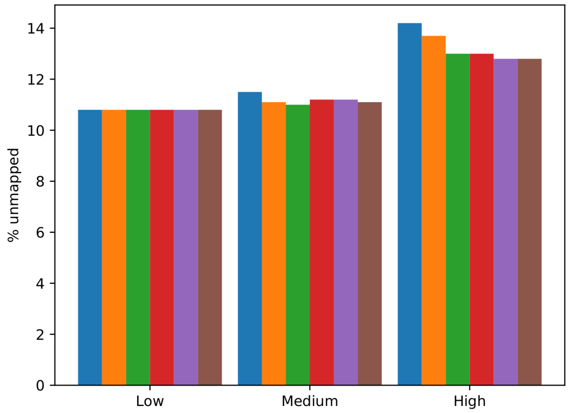

Fig F Percentage of unmapped reads – simulated datasets. The percentage of unmapped reads is plotted for two simulated read datasets mapped to their reference sequences. Results are shown for low, medium, and high compression. **(A)** PacBio reads simulated from the CHM13 ChrX sequence mapped against ChrX sequences from GRCh38. Window sizes of windowed syncmer-minimap were $w = 13, 77, 175$ for the low, medium, and high compression variants, respectively. For windowed syncmer-winnowmap the window sizes were $w = 14, 75, 170$, respectively. **(B)** 1000 ONT reads simulated from CHM13 mapped against GRCh38. Window sizes of windowed syncmer-minimap were $w = 13, 75, 175$ for the low, medium, and high compression variants, respectively and $w = 13, 75, 170$ for the corresponding windowed syncmer-winnowmap variants.

(A) PacBio reads mapped to CHM13X

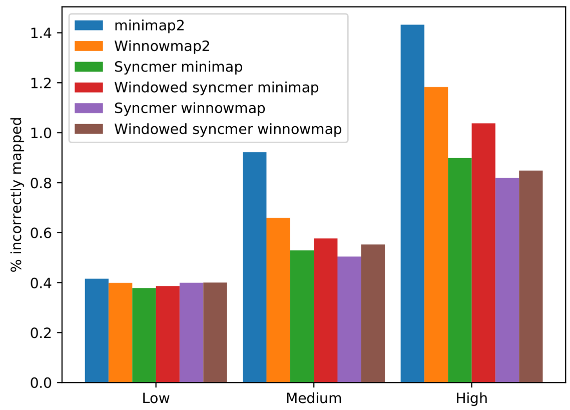

(B) PacBio reads mapped to BAC

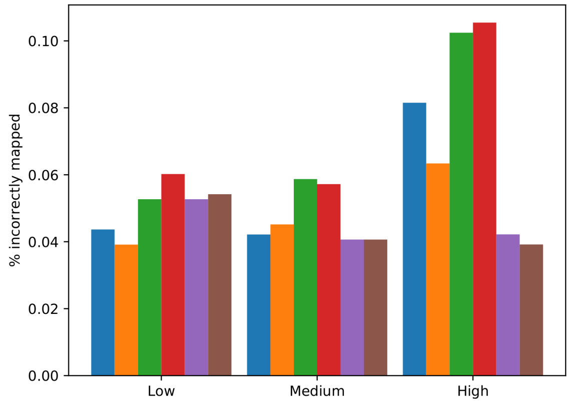

Fig G Percentage of incorrectly mapped reads – simulated data. The percentage of incorrectly mapped reads is plotted for two simulated read datasets and their reference sequences, for mappers using low, medium, and high compression. **(A)** PacBio reads simulated from the CHM13 ChrX sequence mapped against CHM13X. Window sizes of windowed syncmer-minimap were $w = 13, 80, 165$ for the low, medium, and high compression variants, respectively. For windowed syncmer-winnowmap they were $w = 14, 75, 170$, respectively. **(B)** PacBio reads simulated from the 15 bacterial species in BAC mapped against the union of their references. Window sizes of windowed syncmer-minimap were $w = 13, 75, 175$ and in windowed syncmer-winnowmap they were $w = 16, 75, 170$ for the low, medium, and high compression variants, respectively.

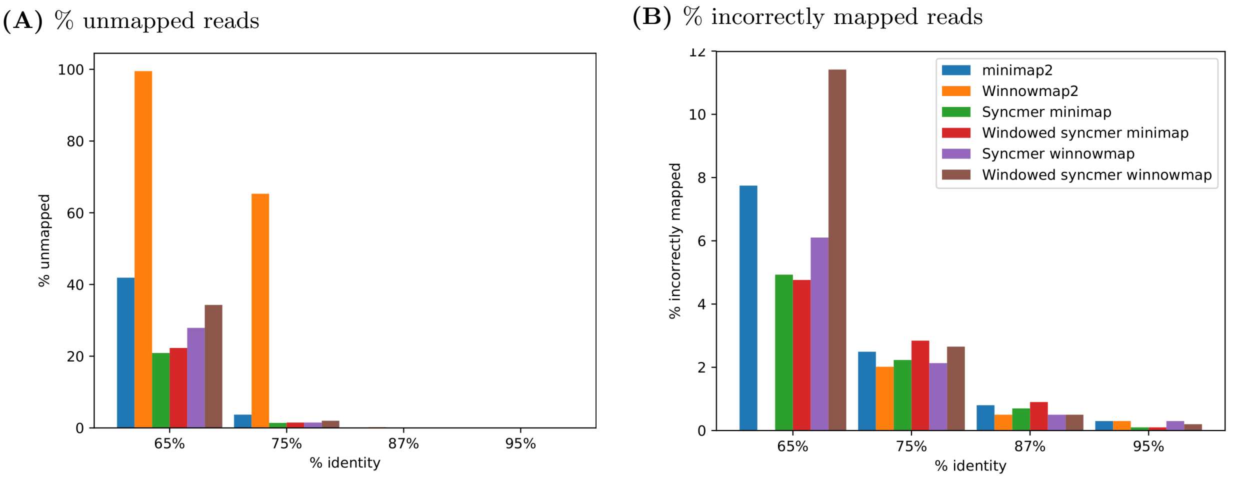

Fig H Impact of percent sequence identity. We varied the mutation rate of 1000 PacBio simulated reads from CHM13X. The figures present the % unmapped and incorrectly mapped for each of the tools. **(A)** % unmapped reads. **(B)** % of the mapped reads that were incorrectly mapped.

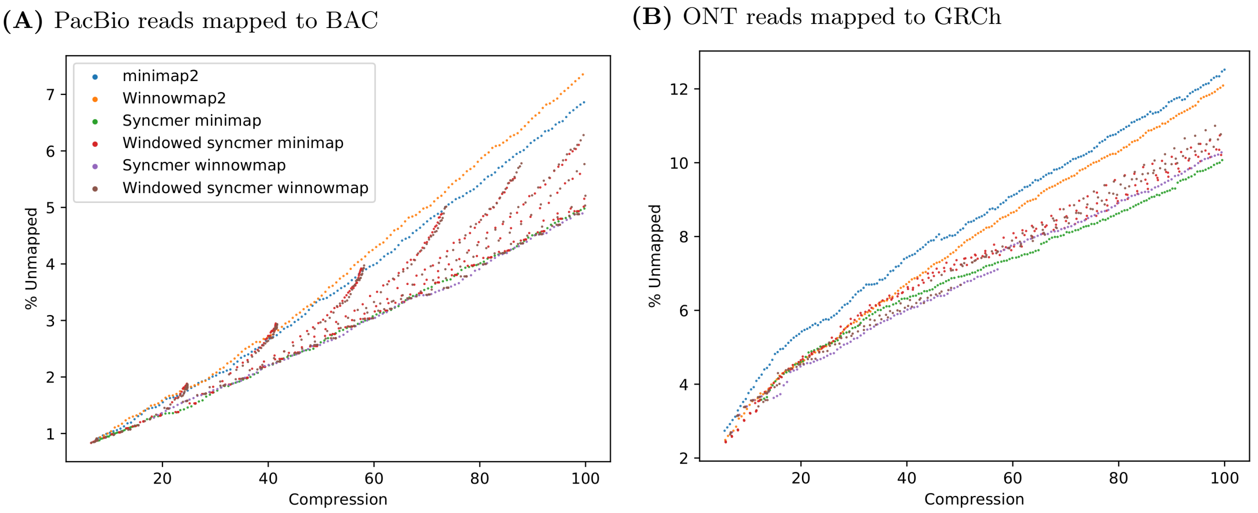

Fig I Percentage of unmapped reads – real datasets. Results are shown across a broad range of compression rates. **(A)** Pooled PacBio bacterial reads. **(B)** ONT human cell-line reads.

variants is presented in Table G.

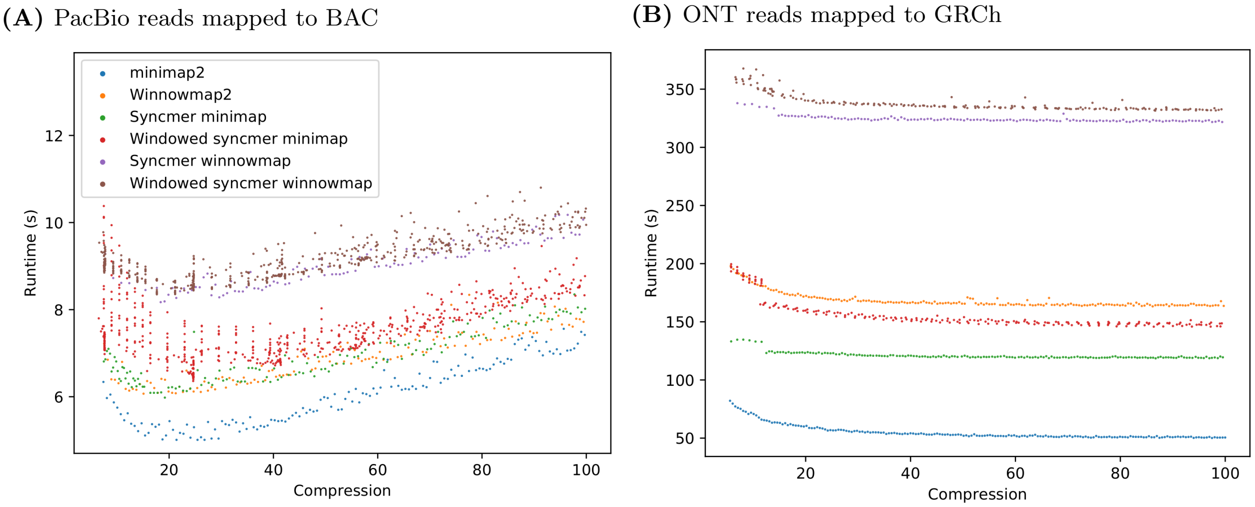

Fig J Runtime vs. compression – real data. The figures show runtime in seconds to index the reference and map reads by each method. **(A)** PacBio bacterial reads. **(B)** ONT human cell-line reads.

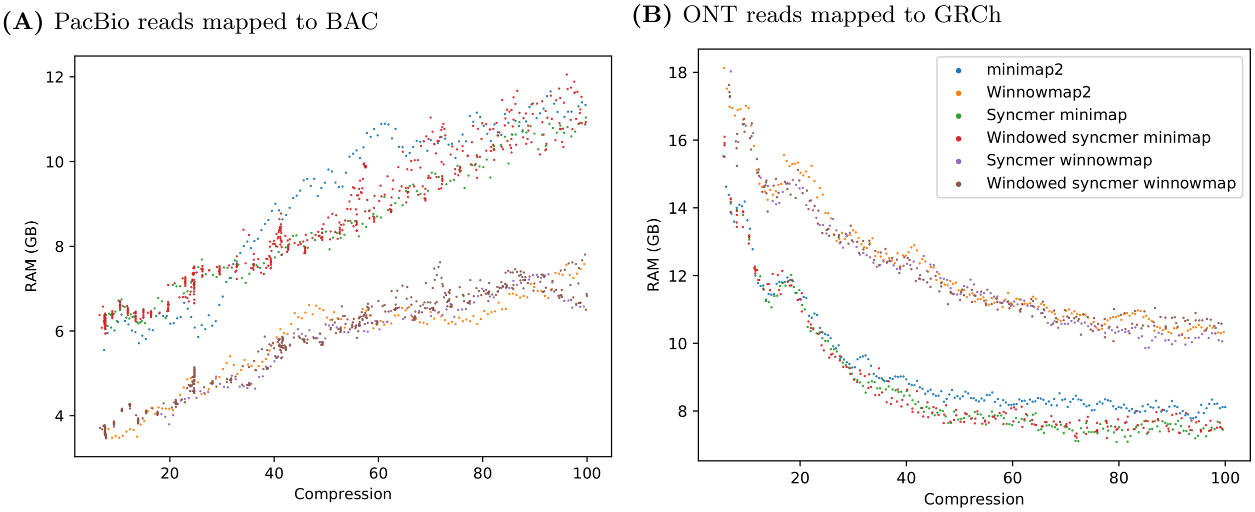

Fig K Memory usage vs. compression – real data. Peak RAM usage in GB to index the reference and map reads for the different methods. **(A)** PacBio bacterial reads. **(B)** ONT human cell-line reads.

Task	Method	Scheme	Index time	Index mem	Map time	Map mem
pbsim bac vs BAC	Windowed syncmer minimap	$\mathbb{S}_{15,5}(3,9)$	4.36	0.32	11.96	2.92
pbsim chm13x vs CHM13X	Windowed syncmer minimap<br>Windowed syncmer minimap	$\mathbb{S}_{15,5}(3,9)$<br>$\mathbb{S}_{15,4}(6)$	11.46<br>7.79	1.01<br>0.45	104.31<br>48.81	5.45<br>9.60
pbsim bac vs CHM13X	Windowed syncmer minimap	$\mathbb{S}_{15,5}(3,9)$	As above	As above	26.10	9.18

Table G Runtime and memory. Time (in seconds) and RAM (in GB) needed to index the reference and map reads by each of the tools. The second and third dataset use the same reference and therefore have the same indexing results. For $\mathbb{S}_{15,5}(3,9)$ window size $w = 13$ was used, with downsampling rate of 1.08 for BAC and downsampling rate of 1.05 for CHM13X. For $\mathbb{S}_{15,4}(6)$ the standard PSS used downsampling rate of 4.13, and the windowed PSS had window size $w = 165$ and downsampling rate 4.37.

References

1. Shaw J, Yu YW. Theory of local k-mer selection with applications to long-read alignment. Bioinformatics. 2021;doi:10.1093/bioinformatics/btab790.

2. Ono Y, Asai K, Hamada M. PBSIM: PacBio reads simulator—toward accurate genome assembly. Bioinformatics. 2013;29(1):119–121.

3. Dohm JC, Peters P, Stralis-Pavese N, Himmelbauer H. Benchmarking of long-read correction methods. NAR Genomics and Bioinformatics. 2020;2(2):lqaa037.

4. Yang C, Chu J, Warren RL, Birol I. NanoSim: nanopore sequence read simulator based on statistical characterization. GigaScience. 2017;6(4):gix010.

5. Jain C, Rhie A, Zhang H, Chu C, Walenz BP, Koren S, et al. Weighted minimizer sampling improves long read mapping. Bioinformatics. 2020;36(Supplement_1):i111–i118.

6. PacificBiosciences. Microbial Multiplexing Data Set 48 plex: PacBio Sequel II System, Chemistry v2.0, SMRT Link v8.0 Analysis; 2019. `https://github.com/PacificBiosciences/DevNet/wiki/Microbial-Multiplexing-Data-Set---48-plex:-PacBio-Sequel-II-System,-Chemistry-v2.0,-SMRT-Link-v8.0-Analysis`.
